# GATR-3, a Peptide That Eradicates Preformed Biofilms of Multidrug-Resistant *Acinetobacter baumannii*

**DOI:** 10.3390/antibiotics13010039

**Published:** 2023-12-31

**Authors:** Monique L. van Hoek, Fahad M. Alsaab, Ashley M. Carpenter

**Affiliations:** 1 Center for Infectious Disease Research, George Mason University, Manassas, VA 20110, USA; 2 School of Systems Biology, George Mason University, Manassas, VA 20110, USA; 3 College of Applied Medical Sciences, King Saud bin Abdulaziz University for Health Sciences, Al Ahsa 36428, Saudi Arabia

**Keywords:** MDR, multidrug-resistant *Acinetobacter baumannii*, biofilm, peptide, polymyxin, antimicrobial peptide, antibiofilm, eradication

## Abstract

*Acinetobacter baumannii* is a gram-negative bacterium that causes hospital-acquired and opportunistic infections, resulting in pneumonia, sepsis, and severe wound infections that can be difficult to treat due to antimicrobial resistance and the formation of biofilms. There is an urgent need to develop novel antimicrobials to tackle the rapid increase in antimicrobial resistance, and antimicrobial peptides (AMPs) represent an additional class of potential agents with direct antimicrobial and/or host-defense activating activities. In this study, we present GATR-3, a synthetic, designed AMP that was modified from a cryptic peptide discovered in American alligator, as our lead peptide to target multidrug-resistant (MDR) *A. baumannii*. Antimicrobial susceptibility testing and antibiofilm assays were performed to assess GATR-3 against a panel of 8 MDR *A. baumannii* strains, including AB5075 and some clinical strains. The GATR-3 mechanism of action was determined to be via loss of membrane integrity as measured by DiSC_3_(5) and ethidium bromide assays. GATR-3 exhibited potent antimicrobial activity against all tested multidrug-resistant *A. baumannii* strains with rapid killing. Biofilms are difficult to treat and eradicate. Excitingly, GATR-3 inhibited biofilm formation and, more importantly, eradicated preformed biofilms of MDR *A. baumannii* AB5075, as evidenced by MBEC assays and scanning electron micrographs. GATR3 did not induce resistance in MDR *A. baumannii*, unlike colistin. Additionally, the toxicity of GATR-3 was evaluated using human red blood cells, HepG2 cells, and waxworms using hemolysis and MTT assays. GATR-3 demonstrated little to no cytotoxicity against HepG2 and red blood cells, even at 100 μg/mL. GATR-3 injection showed little toxicity in the waxworm model, resulting in a 90% survival rate. The therapeutic index of GATR-3 was estimated (based on the HC_50_/MIC against human RBCs) to be 1250. Overall, GATR-3 is a promising candidate to advance to preclinical testing to potentially treat MDR *A. baumannii* infections.

## 1. Introduction

The emergence of multidrug-resistant (MDR) bacteria was first identified in 1955 [1], and the phenomenon continued to persist [2]. One of the most common MDR pathogens is *Acinetobacter baumannii* [3]. *A. baumannii* is a gram-negative bacillus that is aerobic and pleomorphic and causes opportunistic infections in humans [4]. According to the Centers for Disease Control and Prevention, carbapenem-resistant *A. baumannii* caused 8500 infections and 700 deaths in the United States in 2017 [5]. The ability of this bacterium to acquire drug resistance through horizontal gene transfer and potentially exhibit phenotypic resistance within biofilms poses an even greater concern [3]. Immunocompromised individuals, such as severely injured soldiers and burn victims, are susceptible to acquiring *A. baumannii* infections [6]. The distinctive ability of *A. baumannii* to survive desiccation may facilitate bacterial colonization of hospital devices, leading to nosocomial infections [7], particularly human urinary tract infections and wound infections [3]. *A. baumannii* poses a substantial risk for colonizing combat-related injuries with a recalcitrant biofilm-forming infection [8]. Severe wounds caused by traumatic injuries on the battlefield are at significant risk of bacterial contamination from the soil environment or from nosocomial contamination along the combat casualty care chain [9]. Most of the bacterial species that infect such wounds can form biofilms, which exacerbates the prognosis by prolonging the infection, shielding the bacteria from antibiotics and the immune system, and inhibiting wound healing [10,11]. Biofilms are a complex community of single or multiple species of bacteria that are enclosed with extracellular polymeric substances (EPSs), which provide protection from external compounds such antimicrobial agents [12]. Another vulnerable wound site for *A. baumannii* infection is diabetic foot ulcers [13], which affect a significant number of patients with advanced Type 2 diabetes. ESKAPE pathogens (the first letter of each of the following organisms: *Enterococcus faecium*, *Staphylococcus aureus*, *Klebsiella pneumoniae*, *Acinetobacter baumannii*, *Pseudomonas aeruginosa*, and *Enterobacter* species) are common in diabetic foot ulcer infections [14,15,16] where *A. baumannii* accounts for 33% of the bacterial isolates from these infections [17]. When multidrug-resistant (MDR) organisms are identified in diabetic foot infections, they severely influence patient outcomes and the recurrence of infection [18]. Discovering novel therapeutics that are antibacterial against gram-negative MDR organisms and are also effective against these bacteria in biofilms could be a pivotal advancement in treating infected wounds in both military and civilian patients.

Antimicrobial resistance against antibiotics has increased the urgency of developing therapeutic alternatives or cotreatments such as antimicrobial peptides (AMPs). AMPs consist of a polypeptide chain of short lengths (typically 10–50 aa) that possesses a positive net charge and often high hydrophobicity [19]. They have broad-spectrum antimicrobial activity and tend to form secondary structures such as alpha-helical, beta-sheet, extended, and loop conformations [19]. Their broad-spectrum antimicrobial activity and direct action make them potential candidates for therapeutic applications [20].

In this work, we characterized the ability of the GATR-3 peptide to be antimicrobial and antibiofilm against MDR ESKAPE pathogens. We determined GATR-3′s antibacterial and antibiofilm properties against MDR *A. baumannii* using MIC, MBIC, and MBEC assays and scanning electron microscopy. We found that this peptide shows very low host-directed toxicity by multiple measures and has a robust therapeutic index, showing promise as a potential therapeutic for MDR *A. baumannii* and ESKAPE pathogen infections.

## 2. Results

### 2.1. GATR-3 Peptide Design

GATR-3 is a designed peptide inspired by the short Apo6 peptide we previously identified through our BioProspecting study of American Alligator serum [21]. Cryptic AMPs are derived by proteolysis from a large protein that does not itself have antimicrobial activity [22]. Several antimicrobial peptides reported in the literature that have activity against MDR bacteria are cryptic peptides. Another cryptic peptide is the short peptide Apo6 we identified in our BioProspector study derived from apolipoprotein E of the American Alligator [23,24]. The two short Apo peptides represent the peptides cleaved from the C-terminal helix of apolipoprotein E by an endogenous protease that we captured and identified with our BioProspector particles. We found that these two peptides had antibacterial activity against *S. aureus*, *B. ceres*, *E. coli*, *P. aeruginosa*, and *A. baumannii* [23,24]. We further developed these peptides into GATR-3 peptides by modifying the peptide sequence to optimize its amphipathicity and net charge [25]. The sequence and properties of GATR-3 are summarized in Table 1 and were designed to improve the overall amphipathicity, improve the hydrophobic face, and increase the net positive charge. The 22 amino acid peptide is predicted to be mainly helical when analyzed via I-Tasser [26], a structure that is similar to the parental Apo6 peptide [24].

### 2.2. Antimicrobial Susceptibility Testing

Antimicrobial susceptibility testing was performed for GATR-3 against a panel of eight *A. baumannii* strains listed in Table S1 according to the CLSI protocol with the adaptation for peptide testing [28], which eliminates the additional cations. We found that the minimum inhibitory concentration (MIC) of GATR-3 against *A. baumannii* AB5075 [29] was 4 µg/mL. This level of antibacterial activity is within the range of many well-known antibiotics, suggesting that GATR-3 is an effective antibacterial agent. Given the molecular weight of GATR-3 of 2859.354 Da, this is equivalent to 1.4 µM for GATR-3 MIC against *A. baumannii*. GATR-3 exhibited a strong MIC value of 4 µg/mL (Table 2 and Figure S1) against all tested strains in the panel of strains, including MDR *A. baumannii* strains BAA-1605, BAA-1710, BAA-1794, BAA-1795, BAA-1797, BAA-1799, and BAA-1800, some of which were clinical isolates. The control antibiotic polymyxin B had an MIC of 0.25–1 µg/mL, as previously reported [30,31]. The minimum bactericidal concentration (MBC) was determined to be 4 µg/mL after plating each well, which showed no growth in the MIC assay on an agar plate, suggesting that GATR-3 is bactericidal at the MIC. Polymyxin B showed an MBC of 0.5 µg/mL, which is also similar to its MIC.

GATR-3 also showed significant antibacterial activity against MDR ESKAPE pathogens other than *A. baumannii*, including an MIC of 8 µg/mL against *P. aeruginosa* BAA-2110 and *K. pneumoniae* BAA-1705 and 32 µg/mL against *E. asburiae* BAA-3043. The two gram-positive bacteria, *S. aureus* 33592 and *E. faecalis* 51299, were more resistant to GATR-3 than gram-negative bacteria, with an MIC of 64 µg/mL (Table 2). Amikacin, colistin, vancomycin, and levofloxacin were used as control antibiotics for the MIC assay (Table 2), which was performed as expected [32,33].

In conclusion, the GATR-3 peptide is less effective against gram-positive bacteria but represents a highly effective antimicrobial peptide against a wide range of gram-negative MDR ESKAPE pathogens.

### 2.3. Inhibition of Biofilm Formation (MBIC_50_)

Bacterial biofilms are difficult to eradicate, contribute to the aggravation of wound infections, and have limited therapeutic options [34,35,36]. We tested GATR-3 activity on the inhibition of biofilm formation of seven strains of *A. baumannii* (AB5075, BAA-1794, -1710, -1795, -1797, -1605 and -1800) (Figure S2). It has been previously noted that different strains produce different biofilm masses [37,38], which is also what we found. GATR-3 inhibited biofilm formation against all MDR *A. baumannii* strains (Table 3), resulting in an MBIC_50_ of 0.18–21.7 µg/mL when biofilm mass was quantified by crystal violet. Since the biofilm inhibition effect occurred at a concentration that is very close to the growth-inhibitory concentration (data not shown), we conclude that the antibiofilm activity is due to the killing action of the peptide and not due to some direct antibiofilm effect on the bacteria, as has been shown for other peptides such as LL-37 [39,40,41]. The control peptides LL-37 and IDR-1018 showed strain-dependent antibiofilm activity (Table 3), with LL-37 being the most active control (MBEC_50_ 0.31–>64 µg/mL). IDR-1018 showed a remarkable biofilm-inhibitory effect against *A. baumannii* BAA-1797 (Table 3), in agreement with previously published reports [31,42]. Polymyxin B demonstrated antibiofilm activity at antibacterial concentrations across all tested strains, with an MBIC_50_ range of 0.08–1.21 µg/mL (Table 3), as previously published [31,43]. Detailed MBIC results of GATR-3 against all the tested strains can be found in Figure S2. GATR-3 also exhibited dose-dependent inhibition of biofilms against *P. aeruginosa* BAA-2110, *K. pneumoniae* BAA-1705, and *E. faecalis* 51299 (Figure S3A,B,D), while *E. asburiae* BAA-3043 did not form sufficient biofilms for any inhibition effect to be recognized (Figure S3C). GATR-3 was not able to inhibit *S. aureus* 33592 biofilms at the highest concentration tested (64 µg/mL).

Thus, GATR-3 is an effective inhibitor of biofilm formation against gram-negative MDR ESKAPE pathogens, including *A. baumannii*, *P. aeruginosa*, *K. pneumoniae,* and *E. faecalis*, which could be a very useful property in the topical treatment of wound infections.

### 2.4. Eradication of Preformed Biofilms (MBEC)

Since most bacterial biofilms are already formed by the time a disease state is diagnosed, we tested the activity of GATR-3 peptide against preformed biofilms of *A. baumannii* to better assess its potential for treating biofilm-mediated infections in the clinical setting [44]. We evaluated the peptides for their ability to eradicate established (preformed) biofilms in tryptic soy broth for 24 h at 37 °C (for establishing biofilm and drug challenge) using the MBEC (Calgary device) method and crystal violet staining [31,44,45]. The lead peptide GATR-3 showed biofilm-eradicating activity towards seven out of eight tested strains of MDR *A. baumannii*, resulting in an MBEC_50_ as low as 4.1 µg/mL (Table 4). The one outlier, strain BAA-1795, did not form a significant amount of biofilm overall in these experiments and thus also did not show much inhibition (Figure S4). The range of GATR-3 MBEC_50_ was 4.1–9.6 µg/mL, while LL-37 showed a higher range of 2.97–64 µg/mL. LL-37 was surprisingly effective in the MBEC_50_ assays. We and others have previously demonstrated that LL-37 degrades established biofilms of *P. aeruginosa* at 1 µg/mL [40,41]. LL-37 has also been demonstrated to disperse biofilms of multidrug- and pandrug-resistant *A. baumannii* at 64 µg/mL [45,46].

IDR-1018, a peptide that has antibiofilm activity against *P. aeruginosa*, *A. baumannii*, *E. coli*, *K. pneumoniae*, *S. aureus*, *Burkholderia cenocepacia*, and *Salmonella enterica* [42], failed to demonstrate an MBEC_50_ against this organism, as it eradicated only 10–20% of *A. baumannii* biofilms, consistent with our previous experiments [31]. The control, which was cyclic peptide antibiotic polymyxin B, was potent in eradicating preformed biofilms (MBEC_50_ range 0.17–9 µg/mL), as expected since these strains are not colistin or polymyxin B resistant. Detailed MBEC assay results of GATR-3 against *A. baumannii* are shown in Figure S4. The dose-dependent biofilm-eradicating activity of GATR-3 (Figure S4A) was not observed for another highly bactericidal peptide we designed against gram-negative bacteria, HRZN-15 (Table S2), which eradicated biofilms at higher concentrations [31]. The antibiofilm activity profile of GATR-3 surpassed that of a different recently published peptide, SAAP-148 (Table S2), which inhibited and eradicated MDR *A. baumannii* biofilms at 41.3 (12.8 µM) and 82.6 µg/mL (25.6 µM), respectively [47], approximately 10-fold higher than GATR-3 MBEC_50_. SAAP-148 has been proposed for topical application against antibiotic-resistant bacteria rabbits and human skin models [47], suggesting that GATR-3 might also be a candidate for such an approach.

In conclusion, the GATR-3 peptide is effective at eradicating preformed biofilms of almost all of the strains of MDR *A. baumannii* that we tested, with the exception of *A. baumannii* BAA-1795.

**Figure 1:**
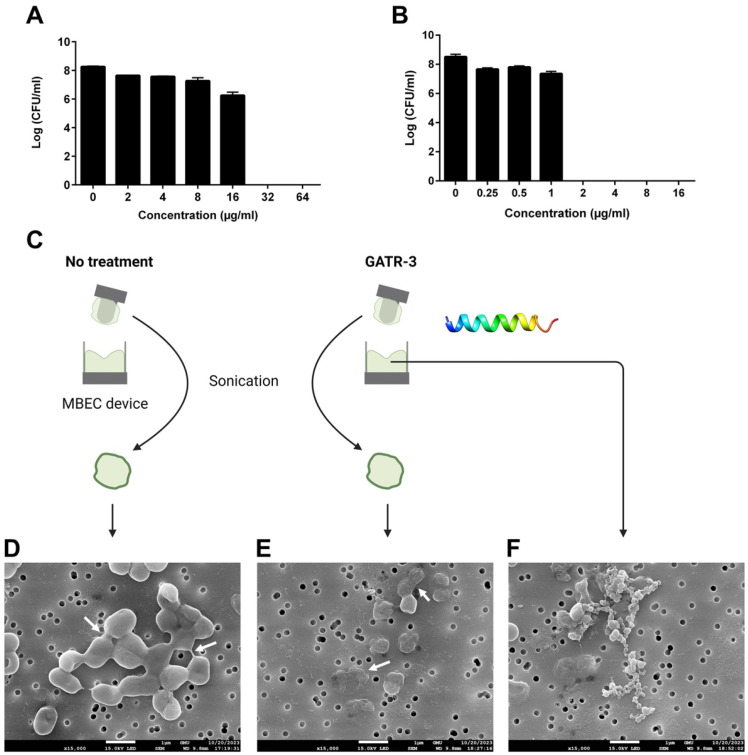
Log reduction in CFUs of AB5075 using a minimum biofilm eradication concentration (MBEC) device. Preformed biofilms of AB5075 were challenged with (**A**) GATR-3 and (**B**) polymyxin B (*n* = 3), which resulted in complete eradication of the bacteria at 32 and 2 µg/mL, respectively. The figures represent the number of bacteria present in biofilms at each tested concentration. The detailed mean and standard deviation values can be found in Table S3. (**C**) Schematic diagram of samples collected for SEM imaging. (**D**–**F**) Scanning electron micrographs of the *A. baumannii* AB5075 biofilm. Bacteria were grown on pegs using an MBEC device to establish biofilms. After incubation, biofilms were treated with 32 µg/mL GATR-3. (**D**) Untreated bacteria (white arrows indicate the slimy material) and (**E**) GATR-3-treated bacteria (white arrows indicate dead cells) were retrieved after sonication of the pegs. (**F**) GATR-3-treated bacteria in the treatment well (detached from the pegs before sonication). Images are taken at 15,000× with a working distance of 9.8 mm. The white bar represents 1 µm.

To further elucidate the effect of GATR-3 on the bacteria present in biofilms, colony-forming unit (CFU) log reduction within the biofilm was determined using an MBEC device with *A. baumannii* strain AB5075 (Figure 1) [44]. This strain was selected because it has been proposed as an MDR model strain of *A. baumannii* for studying pathogenesis and antimicrobial testing [29]. In this method, GATR-3-treated and untreated pegs were gently sonicated to dislodge biofilms. Then, the samples were serially diluted and spotted on enriched agar, and CFUs were counted. GATR-3 completely eradicated MDR *A. baumannii* AB5075 preformed biofilms at 32 µg/mL (Figure 1A), while there was still biofilm observed at 16 µg/mL (Figure S5). While this concentration is higher than the MIC (4 µg/mL) and the MBC (also 4 µg/mL), it is only 8-fold higher and likely reflects the “killing of bacteria” as the mechanism by which GATR-3 is able to eradicate the preformed biofilm. We conclude this because the biofilm on the MBEC device was dislodged and cultured on tryptic soy agar, and bacteria did not grow back in that experiment. We also confirmed this finding by dislodging the biofilm and re-growing it in tryptic soy broth for 24 h, and it also did not show any growth. Polymyxin B was also used as a control, and it exhibited an MBEC of 2 µg/mL (Figure 1B), in agreement with our previous results [31]. Therefore, the mechanism of biofilm eradication is through killing the bacteria.

Samples from the MBEC device (with *A. baumannii* AB5075) were visualized under a scanning electron microscope (SEM). In this experiment, bacteria were grown on pegs to establish biofilms and treated the next day with 32 µg/mL GATR-3. Samples were sonicated to dislodge biofilms from the pegs (Figure 1C) [44]. The untreated sample showed bacteria encased in a smooth regular structure linking the cells together in aggregates covered in a slimy material, as indicated by the white arrows (Figure 1D), which likely represents the biofilm. In contrast, GATR-3-treated samples demonstrated significant damage to bacteria and biofilms, resulting in shrinkage of cells, disruption of the bacterial membrane, a decrease in the number of cells, and loss of the interconnected material (Figure 1E). We also observed accumulated cell debris (Figure 1F) in the well of the GATR-3-treated sample (material that detached from the pegs due to peptide treatment before sonication). The debris included round objects that were much smaller than the bacteria, as seen in relation to the filter pores in the image (Figure 1F). We also examined the subbiofilm eradication concentration (16 µg/mL) of GATR-3 in the treatment well. Compared to Figure 1F, the sample treated with 16 µg/mL GATR-3 shows bacterial biofilm indicated by the sheet of EPS with attached and embedded bacterial cells (Figure S5).

### 2.5. Time to Kill Assay

**Figure 2:**
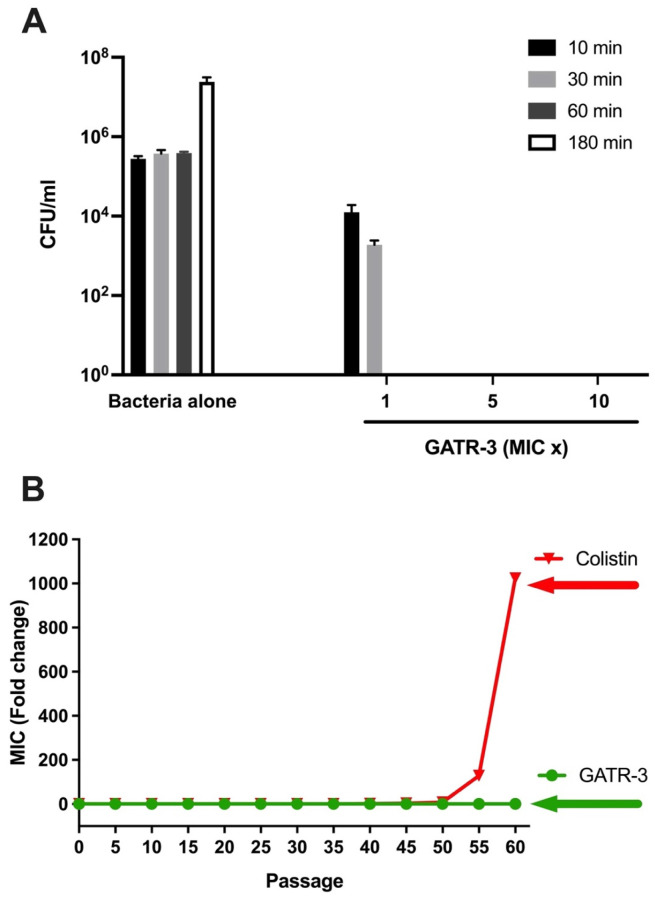
Time to Kill and Resistance of GATR-3 peptide. (**A**) Time-kill kinetics of GATR-3 against *A. baumannii* AB5075 upon exposure to 1-, 5- and 10-fold the MIC (4 µg/mL) over 10, 30, 60, and 180 min. “MIC ×” indicates the multiple of MIC used. (**B**) Resistance acquisition of AB5075 to GATR-3 and colistin was performed upon exposure to subinhibitory concentrations of the challenge.

To assess the killing kinetics of GATR-3, MDR *A. baumannii* AB5075 was co-incubated with GATR-3 at 1-, 5- and 10-fold MICs, and the survival of bacteria was determined at 10-, 30-, 60- and 180-min time points (Figure 2A) [48,49]. At the MIC concentration (4 µg/mL), GATR-3 completely killed *A. baumannii* within 1 h. However, the peptide showed very fast killing activity at 5- and 10-fold MIC, eradicating the bacteria within 10 min. These results reflect the antimicrobial activity of the peptide against planktonic *A. baumannii*. This fast-killing activity is similar to what has been observed with the ZY4 peptide [50]. GATR-3 killing time was faster than that of our previously reported peptide, HRZN-15, against this organism [31].

### 2.6. Induction of Resistance by GATR-3 Peptide

The propensity of GATR-3 to induce resistance in *A. baumannii* was assessed by challenging the bacteria with subinhibitory concentrations of GATR-3 and passing the bacteria after 24 h of incubation [51]. AB5075 demonstrated no resistance against GATR-3 even after 60 days of challenge (Figure 2B). In contrast, the bacteria gained significant resistance to colistin on the 40th day (resistant to 2 MIC) (Figure 2B). *A. baumannii* acquired resistance to colistin when the drug concentration was increased, starting at passage 31. The colistin-exposed bacteria continued to increase resistance (resistant to 1024-MIC fold) on the 60th day. The reason that *A. baumannii* gained resistance on the 40th day in this study could be that bacteria were challenged with a lower concentration of colistin than in our previous study, where we observed resistance on the 15th day [31]. Mwangi et al., also previously demonstrated that *A. baumannii* developed resistance to colistin within 15 days of exposure [50], although different strains were used.

Evaluation of potential cross-resistance of colistin-resistant *A. baumannii* to GATR-3 was assessed [51]. Colistin-resistant *A. baumannii* generated in this study was as sensitive to GATR-3 as the wild type (MIC of 4–8 µg/mL) (Figure S6A). The colistin resistance of this organism was confirmed (Figure S6B). These findings suggest that GATR-3 has a low ability to induce resistance and is not subject to cross-resistance to colistin.

### 2.7. Mechanism of Action

As suggested from the previous results, GATR-3 was able to effectively inhibit the growth of planktonic *A. baumannii* as well as kill the bacteria within preformed biofilms. Therefore, we wanted to determine how the peptide acts on bacteria. Antimicrobial peptides such as LL-37 [52], HRZN-15 [31], and magainin-2 [53] induce cell death by forming pores on the bacterial membrane. To assess the mechanism of action of GATR-3, two assays were performed to explore whether the peptide can depolarize and/or disrupt the bacterial membrane.

**Figure 3:**
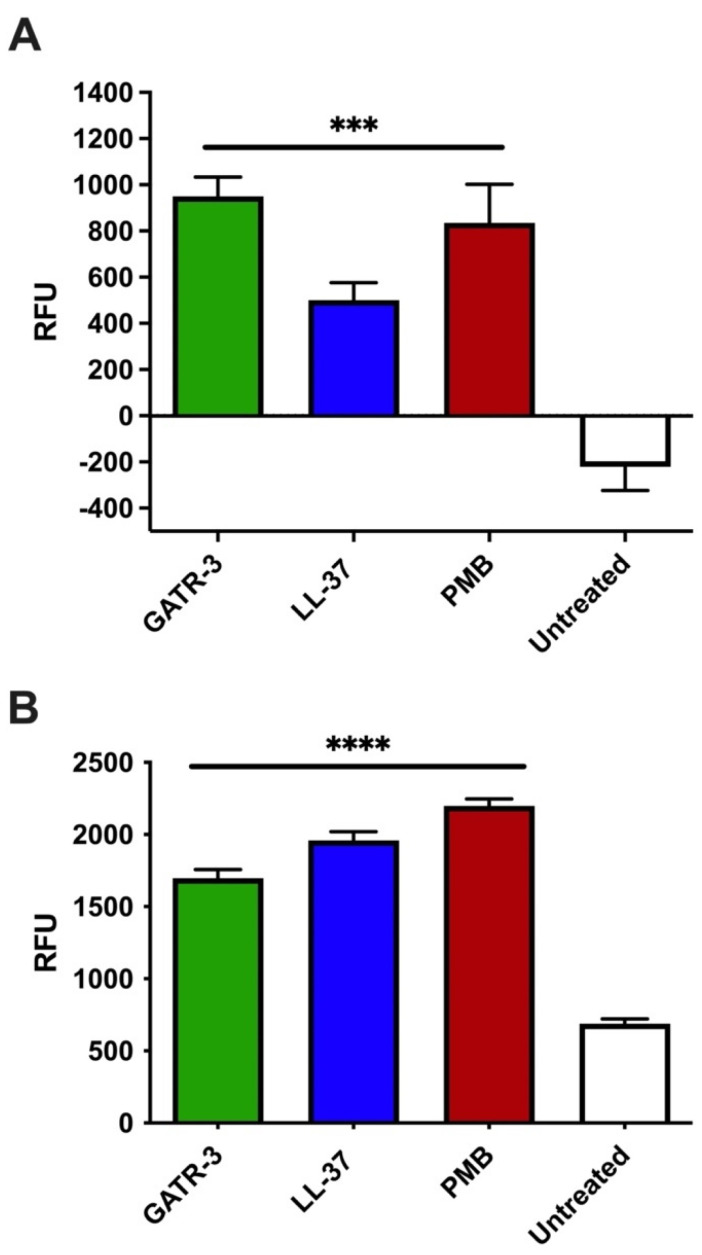
Membrane effects of GATR-3 on MDR *A. baumannii* AB5075. (**A**) Membrane depolarization by peptides GATR-3, LL-37, and polymyxin B (PMB) as assessed by the fluorescent dye DiSC_3_(5). (**B**) Membrane disruption (pore formation) was also assessed by ethidium bromide. Bacteria were incubated with 50 µg/mL peptide/drug, and the results were obtained after 20 min of challenge exposure. Student’s *t*-test was performed on untreated against treated bacteria (*** *p* < 0.001, **** *p* < 0.0001). The experiment was repeated twice, and one representative experiment was chosen.

Some peptides, such as cell-penetrating peptides, do not have effects on the bacterial membrane but can penetrate and have only intracellular targets. As a measure of the disturbance of the bacterial membrane by GATR-3, we measured both depolarization and ethidium bromide staining. Depolarization indicates transient cytoplasmic membrane disruption that allows for ion leakage, which then damages the proton motive force. We utilized a cationic fluorescent dye, 3,3′-dipropylthiadicarbocyanine iodide (DiSC_3_(5)), to assess the depolarization effect of the peptide on the *A. baumannii* membrane. The dye migrates through the bacterial outer membrane, accumulates on the cytoplasmic membrane, and quenches. If the peptide causes an imbalance in the membrane potential, the dye will be released, and fluorescence will be detected. In Figure 3A, GATR-3 depolarized the membrane of AB5075, as evidenced by the fluorescence increase compared to the untreated control. LL-37 and polymyxin B were used as controls, and they also exhibited membrane-depolarizing effects, in agreement with previous reports from our laboratory and others [54,55,56,57,58], with LL-37 showing less depolarization than the other peptides. The kinetics of membrane depolarization were recorded and can be found in Figure S7A.

Bacterial outer membrane permeability is monitored using the ethidium bromide uptake assay, which demonstrates when peptides lead to major, catastrophic disruptions of the bacterial membrane [59]. Since we found that the peptide disrupted the bacterial membrane potential, we further explored the membrane permeabilization effect of the peptide using ethidium bromide (EtBr) [54,55]. EtBr cannot permeate the bacterial membrane when the membrane is intact. If the bacterial membrane integrity is lost (through lysis or pore formation), EtBr intercalates into bacterial DNA, emitting fluorescence. GATR-3 peptide permeabilized the AB5075 membrane (Figure 3B), as indicated by the increase in fluorescence units. We previously demonstrated that LL-37 can cause membrane disruption of *Escherichia coli* and *Francisella novicida* with the same technique [24]. We found that GATR-3 perturbed the membrane of MDR *A. baumannii* and that the control antibiotic polymyxin B and control peptide LL-37 demonstrated similar effects (Figure 3B). Figure S7B demonstrates the quick action of GATR-3, as fluorescence was detected immediately after peptide exposure (time 0).

Thus, the GATR-3 peptide can depolarize and form pores in the bacterial membrane as its mechanism of direct antibacterial action against MDR *A. baumannii*.

### 2.8. Host-Directed Toxicity of GATR-3 Peptide

The host-directed toxicity of the GATR-3 peptide was assessed by three measurements: hemolysis, cytotoxicity, and waxworm toxicity assays.

#### 2.8.1. Hemolysis Assay

**Figure 4:**
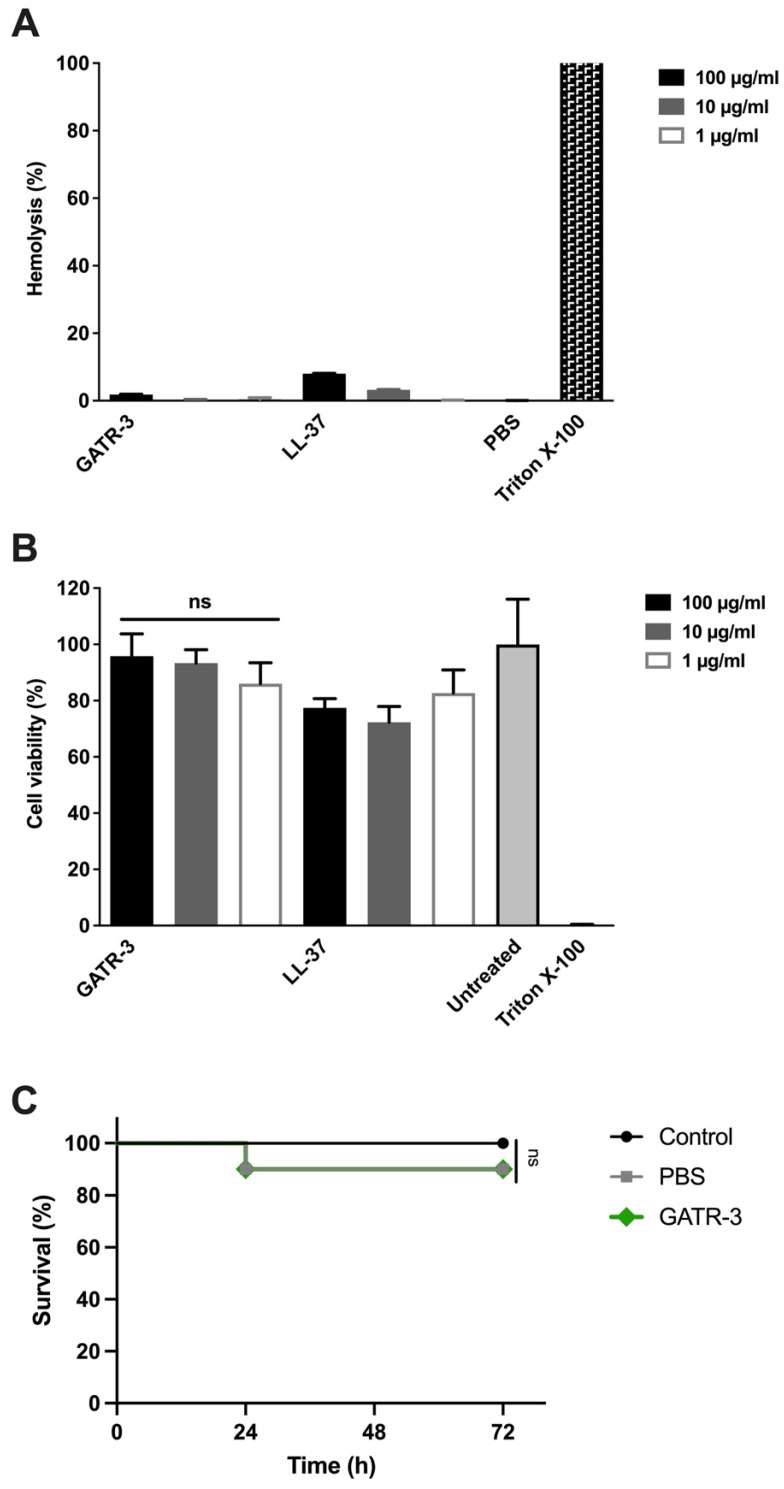
Hemolysis, cytotoxicity, and toxicity assays of GATR-3. (**A**) Hemolytic activity of GATR-3 and LL-37 against human red blood cells. A 2% RBC suspension was incubated with 100, 10, or 1 µg/mL of each peptide for 1 h at 37 °C. The release of hemoglobin (*n* = 3) was measured at OD_540_, and the percentage of hemolysis was calculated relative to the Triton X-100-treated RBCs (100% hemolysis). (**B**) Cell viability of HepG2 cells was determined (*n* = 3) using the MTT assay upon exposure to GATR-3 and LL-37 at 100, 10, and 1 µg/mL. Triton X-100-treated wells and untreated cells were used as 0% and 100% viability controls, respectively. Student’s *t*-test was performed to determine the statistical significance of GATR-3-treated cells compared to untreated cells (ns = not significant). (**C**) The toxicity of GATR-3 was assessed in *Galleria mellonella* (*n* = 10) by injecting 10 µg of the peptide in 10 µL of sterile PBS in the rear right proleg. The uninjected group and the group injected with 10 µL of sterile PBS served as controls. The GATR-3 result is masked by the PBS result. The Mantel–Cox statistical test was performed (ns = not significant).

Hemolysis was performed with defibrinated whole human blood in triplicate, comparing the activity of GATR-3 with the control peptide LL-37, along with “no peptide” controls, Triton X-100, and PBS (Figure 4A). GATR-3 showed no hemolysis at any of the peptide concentrations tested. Low (8%) but measurable hemolysis was observed for LL-37 at a concentration of 100 µg/mL, in agreement with previous reports from our lab and others [24,31,39,60].

#### 2.8.2. Cytotoxicity Assay

Cytotoxicity assays were performed using the MTT assay on peptide-treated HepG2 cells (Figure 4B) [61], which are human hepatocyte carcinoma cells, as an in vitro model of cell toxicity for antibiotics or antimicrobial peptides [62]. Similar to our previous results in other cell types [24,63] and other published results [64], the LL-37 peptide was relatively nontoxic to these cells, showing 77.4% cell viability at 100 µg/mL [65]. We previously showed that the related first-generation peptides Apo5 and Apo6 were nontoxic to A549 cells [24] at 100 µg/mL. Here, we show that GATR-3 demonstrates 95.8% cell viability at 100 µg/mL.

#### 2.8.3. Waxworm Toxicity Assay

The toxicity testing of GATR-3 was further evaluated using an in vivo *Galleria mellonella* model as previously described [31]. The whole organism toxicity of GATR-3 was further evaluated using an in vivo *Galleria mellonella* model, as we have performed previously [31]. Each larva was injected with 10 µg of GATR-3 dissolved in 10 µL of PBS (1 mg/mL) into one of the rear prolegs, and survival was monitored for 3 days. The GATR-3-treated group demonstrated 90% survival (Figure 4C), which was comparable to that of the PBS-treated group. The control group, which was not injected, maintained 100% survival (Figure 4C). The Mantel–Cox statistical analysis test was performed to compare the control group with the treated groups, and it resulted in no significant difference (*p* = 0.31). This toxicity model has previously been correlated with hemolysis testing when the HRZN-15 peptide produced toxicity in both settings [31]. GATR-3 presents a highly active therapeutic candidate against bacteria with little to no toxicity profile. This waxworm toxicity model correlated well with peptide hemolysis activity when we previously demonstrated that the HRZN-15 peptide produced toxicity in both settings [31]. Thus, we conclude that GATR-3 at an approximate concentration of 1 mg/mL is not toxic to waxworms by systemic injection.

### 2.9. Therapeutic Index

The therapeutic index is a widely used parameter used to characterize and evaluate a peptide’s effect compared to its toxicity [66]. There are two common definitions in the literature for the therapeutic index: the ratio of MHC to MIC [67] (MHC therapeutic index) and the ratio of HC_50_ to MIC (HC_50_ therapeutic index) [68]. MHC is defined as the concentration of peptide inducing 10% hemolysis. For therapeutic purposes, peptides should have a strong interaction with bacteria with little to no interaction with mammalian cells; thus, the higher the therapeutic index is, the greater the antimicrobial specificity, and the more effective and safer the therapeutic. To determine the amount of peptide needed to cause hemolysis, hemolytic activity was determined on defibrinated human red blood cells following our protocol as described above but at higher peptide concentrations. GATR-3 was tested at the following concentrations: 1, 10, 50, 100, 500, 1000, 2500, and 5000 µg/mL to determine the MHC (minimum hemolytic concentration) and HC_50_, the concentration causing 50% lysis of human red blood cells. In the experiments performed (Figure S8), we observed ~50% hemolysis of human blood at 5000 µg/mL (5 mg/mL) GATR-3 peptide. Thus, the HC_50_ concentration was determined to be 5000 µg/mL.

The therapeutic index (TI) was then calculated using two common formulas, summarized in Table 5. For MDR *A. baumannii,* GATR-3 peptide was found to have high efficacy with an MIC of 4 µg/mL, an MHC of 595 µg/mL, and an HC_50_ of 5 mg/mL. Based on the two different formulas, the therapeutic index was calculated to be either 149 (MHC therapeutic index, MHC/MIC) or 1250 (HC_50_ therapeutic index, HC_50_/MIC). In the literature, there is a wide range of what is determined to be an effective or not effective TI. In one study, V13K_L_ was determined to have a TI of 163, while its analog, native peptide V_681_, had a therapeutic index of 1.8 [67]. Bacalum et al. reported a TI range of 177–579 for cecropin A (CA) peptide and a significantly low TI of 1.82 for melittin due to its strong toxic effects. Both of these studies define TI as MHC/MIC and suggest that the higher the TI value is, the more effective the peptide is against bacteria while exhibiting low or no toxic effect for mammalian cells [68]. In another study, TI, defined as HC_50_/MIC, was improved for Piscidin 1 and Dermaseptin S4 against *A. baumannii*. Peptide analogs, D-Piscidin 1 I9K and D-Dermaseptin S4 L7K, A14K, showed dramatic improvement with TIs of 33 and 219, respectively, by reducing peptide toxicity toward mammalian cells [66]. Understanding and determining the TI early is essential to advance peptides through preclinical trials [69], and the TI of GATR-3 is favorable for further development of this peptide.

Overall, GATR-3 represents a highly active therapeutic candidate against bacteria with a favorable toxicity profile.

## 3. Discussion

Antimicrobial resistance is a global crisis that limits our ability to combat threatening bacterial infections. MDR *A. baumannii* has become a significant concern in military and civilian healthcare settings for nosocomial infections, especially of wounds [70,71]. The rising emergence of MDR pathogens such as *A. baumannii* and the limited discovery of new antimicrobial agents to combat them highlight the urgent need for developing novel therapeutics, such as AMPs, to be used in conjunction with antibiotics to promote clearance and healing of such wounds.

In this study, we investigated the antimicrobial properties of a synthetically designed AMP GATR-3 derived from a cryptic peptide from the American alligator (thus the peptide name pronounced “gator”) and characterized its potential to be developed as a therapeutic candidate against *A. baumannii* and other gram-negative MDR ESKAPE pathogens.

GATR-3 exhibits significant antimicrobial activity against gram-negative MDR ESKAPE pathogens, which are notorious for their resistance to conventional antibiotics and for causing nosocomial infections, including *P. aeruginosa* BAA-2110 and *K. pneumoniae* BAA-1705 (MIC = 8 μg/mL). This peptide has lower activity against the MDR gram-negative *E. asburiae* BAA-3043 (32 μg/mL) and interestingly had poor activity (64 μg/mL) against MDR gram-positive ESKAPE pathogens, including *E. faecalis* 51299 and MRSA (*S. aureus* 33592).

GATR-3 demonstrated even more potent antimicrobial activity against MDR *A. baumannii*. Unlike the parent peptide Apo6, which could not achieve complete killing (MIC) against this organism [24], the ability of GATR-3 to kill this bacterium under MIC conditions (MIC = 4 μg/mL) highlights its ability to function in a physiological environment where salts are present (i.e., salt resistant). Notably, GATR-3 showed rapid killing action against MDR *A. baumannii* compared to the antibiotic polymyxin B, highlighting its potential utility in treating infections where prompt bacterial eradication is needed. The MIC of polymyxin B is lower than for GATR-3, but given the significant toxicity of polymyxin B in humans, alternatives are needed. GATR-3′s strong activity against gram-negative ESKAPE pathogens suggests it has the potential to be developed for combating clinically relevant bacterial infections such as burns and wound infections.

The mechanism of action characterization of GATR-3 revealed that it acts at the membrane of *A. baumannii*. The membrane disruption effect occurs through depolarization and permeabilization, leading to loss of bacterial membrane integrity. This mechanism likely contributes to the rapid bactericidal activity of the peptide.

Importantly, GATR-3 showed no propensity to induce resistance against MDR *A. baumannii* in contrast to colistin, which induced a high level of resistance over the passages tested. GATR-3 also demonstrated no cross-resistance to the colistin-resistant strains we generated.

Biofilms are complex matrices that bacteria establish, and they provide a protective niche from the external environment, making bacteria highly resistant to antimicrobial agents [72]. A key attribute of GATR-3 is that it is also able to eradicate preformed biofilms of MDR *A. baumannii*, which is critical to GATR-3′s potential future utility as a topical treatment for MDR ESKAPE pathogen infections of wounds or burns or surgical sites. Our findings indicate that GATR-3 was able to inhibit biofilm production of MDR *A. baumannii* as well as penetrate and eradicate preformed biofilms and effectively kill bacteria residing within the biofilm. This is a significant effect of GATR-3, as biofilms play a critical role in the pathogenesis of chronic wounds and device-related infections [73].

GATR-3 also demonstrated a favorable safety profile, as it did not exhibit lysis or toxicity against human blood, cultured cells, or the waxworm *Galleria mellonella* at high concentrations. We estimated the therapeutic index of GATR-3 to be between 149–1250, which is strongly favorable and suggests that this peptide may be effective against the pathogen while not being toxic to the host. It is less toxic and more effective than the human cathelicidin peptide LL-37.

Compared to other antimicrobial peptides, GATR-3 displays strong antibacterial and antibiofilm properties as well as minimal toxicity. For example, pexiganan (MSI-78) is proposed as a topical treatment for diabetic foot infections, and it is undergoing phase 3 clinical trials [74]. Pexiganan was less active than GATR-3 against biofilms of *P. aeruginosa* and *A. baumannii,* even though growth-inhibitory effects are comparable [74,75,76]. It has been demonstrated that pexiganan exhibits toxicity towards human erythrocytes, which is not observed for GATR-3 at the same concentrations [77]. The human cathelicidin LL-37 has been reported to have antibiofilm activity against *P. aeruginosa* [40,41]. However, we demonstrated that GATR-3 is more effective in its antibiofilm activity than LL-37 against *A. baumannii*. LL-37′s lack of antibiofilm activity for this organism is also supported by Feng et al. [45]. It was recently reported that LL-37 enhanced the healing of mild diabetic foot infections but failed to eradicate bacteria in a human trial [78], suggesting that a peptide with stronger wound-related performance might do better.

The significance of this work is that GATR-3 has very favorable antibacterial and antibiofilm activities and thus far lacks significant toxicity, all of which are essential attributes for further development. Bacterial biofilms are generally highly recalcitrant to systemic treatment and contribute to ongoing inflammation and tissue damage in infected wounds. In particular, the ability to eradicate a preformed biofilm of this dangerous bacterium is critical to GATR-3’s anticipated application in the clinic as a potential topical treatment for military and civilian-infected wounds, likely in combination with antibiotics. This manuscript represents a significant step forward in our preclinical development of the GATR-3 peptide. This manuscript sets the stage for our future in vivo work in which we will demonstrate GATR-3 efficacy against MDR *ESKAPE* infection and lack of toxicity in vivo.

In conclusion, our study presents a synthetic peptide GATR-3 that has antimicrobial activity against clinically relevant, drug-resistant, biofilm-forming, gram-negative bacteria. Its rapid bactericidal action, biofilm-eradication ability, lack of resistance induction, and favorable preliminary safety profile all suggest that it may be a promising candidate for development as a therapeutic for treating bacterial infections.

## 4. Materials and Methods

### 4.1. Bacterial Strains

Multidrug-resistant ESKAPE pathogens were tested, including *Enterococcus* (*E.*) *faecalis* 51299, *S. aureus* 33592, *K. pneumoniae* BAA-1705, *P. aeruginosa* BAA-2110, and *Enterobacter* (*E.*) *asburiae* BAA-3043. A library of multidrug-resistant (MDR) *A. baumannii* strains was used in this study (Table S1). MDR strains BAA-1710, -1794, -1795, -1797, -1799, -1605, and -1800 were obtained from American Type Culture Collection (Manassas, VA, USA). AB5075 (MRSN 959) was obtained from BEI Resources (NR-52248, Manassas, VA, USA), and it is a part of the MDR diversity panel. AB5075 was selected because it has been well-characterized as a model strain for studying pathogenesis and antimicrobial susceptibility testing [29,79]. All bacterial strains were cultured in Tryptic Soy broth (TSB) (BD 211825) in a shaking incubator at 37 °C for 24 h. Bacteria were aliquoted and stored at −80 °C with glycerol (20% final concentration). Bacteria were grown on Tryptic Soy agar before each assay, and three to five colonies showing opaque morphology were selected to be grown for the experiment.

### 4.2. Peptide Synthesis

Peptides were synthesized by Fmoc solid phase synthesis from ChinaPeptides Inc. (Shanghai, China) with high purity ≥ 98%, including GATR-3, IDR-1018, and LL-37. LL-37 from ChinaPeptides Inc. was used for antibacterial assays. For the hemolysis assay, LL-37 was obtained from Direct Peptides Inc. (Orlando, FL, USA) with a high purity of 99% for comparison to the ChinaPeptides product. In all cases, purity was confirmed by reverse-phase high-performance liquid chromatography and ESI-mass spectrometry by the manufacturer (Figures S9–S11). Polymyxin B sulfate (Cat# 5291) was obtained from EMD Millipore Corp (Burlington, MA, USA).

### 4.3. Minimum Inhibitory Concentration (MIC)

Minimum inhibitory concentration was performed according to the Clinical and Laboratory Standards Institute (CLSI). A minor modification to the CLSI protocol was introduced, which is the use of Difco Mueller Hinton broth (BD 275730, Franklin Lakes, NJ, USA) instead of cation-adjusted MHB [80]. Fifty microliters of 1 × 10^6^ CFU/mL (*n* = 3) was challenged with various peptide concentrations (0.25–64 µg/mL) in polypropylene 96-well plates (Corning 3879, Corning, NY, USA) to achieve a final bacterial concentration of 5 × 10^5^ CFU/mL at 37 °C and 5% CO_2_ for 20–24 h in Difco Mueller Hinton broth [28]. Bacterial growth was measured spectrophotometrically at OD_600_ nm. LL-37 and polymyxin B were used as controls [40,41,42,81]. Wells containing MHB alone or MHB with the challenge were used as sterility controls. Wells containing only bacteria served as growth control. The experiment was repeated twice, and one representative was selected and presented.

### 4.4. Minimum Bactericidal Concentration (MBC)

An aliquot of 50 µL of MIC experimental wells that showed no growth was plated on Tryptic Soy agar and incubated for 24 h at 37 °C and 5% CO_2_ as previously described [82]. A peptide concentration that showed no bacterial growth was MBC. The experiment was performed twice.

### 4.5. Time-Kill Kinetics

The time-kill kinetics were assessed as follows, generally following [49]. Fifty microliters of 1 × 10^6^ CFU/mL AB5075 (*n* = 3) was challenged with 50 µL of 1, 5, and 10 MIC folds of GATR-3 or polymyxin B for 10, 30, 60, and 180 min in Difco Mueller Hinton broth (BD 275730) using polypropylene 96-well plate (Corning 3879). At each time point, samples were serially diluted using DPBS (Gibco, Waltham, MA, USA), and colony-forming units (CFU) were determined by spot plating on Tryptic Soy agar [83].

### 4.6. Minimum Biofilm Inhibitory Concentration (MBIC)

Minimum biofilm inhibitory concentration was determined as described previously with minor modifications [40,84] following the standardized methods [36,80]. Briefly, 100 µL of 1 × 10^5^ CFU of bacteria in TSB was incubated with a series of decreasing concentrations of peptide (0.25–64 µg/mL) in a polystyrene 96-well plate (Falcon 353072, Corning, NY, USA) for 24 h at 37 °C. After incubation, bacterial growth was measured at the OD_600_ nm. Spent media was removed, and the wells were washed three times with cold tap water. Biofilm in wells was then heat-fixed at 70 °C for 1 h. An aliquot of 200 µL of 0.1% crystal violet diluted in deionized water was added to each well and incubated for 15 min to allow biofilms to be stained. Excess stain was rinsed with cold tap water, and plates were air-dried. An aliquot of 200 µL of 33% glacial acetic acid was added to each well to resolubilize the crystal violet stain. Biofilm mass was measured spectrophotometrically at OD_590_ nm. Wells containing only bacteria served as 100% biofilm. Media alone and media with the challenge were used as sterility controls as well as 0% biofilm. Polymyxin B, LL-37, and IDR-1018 were used as positive controls [40,41,42,85]. MBIC_50_ values were calculated by fitting the data from the MBIC assay (Figure S1) to a standard sigmoidal dose–response curve.

### 4.7. Minimum Biofilm Eradication Concentration (MBEC)

MBEC was performed to determine the lowest concentration of a compound that eradicates established biofilm using the MBEC assay (Innovotech Cat #1911, Edmonton, AB, Canada) following the manufacturer’s protocol [44,86]. An aliquot measuring 150 µL (*n* = 3) of a bacterial suspension containing 1 × 10^6^ CFU/mL of the AB5075 strain in Tryptic Soy broth underwent incubation for 24 h at 37 °C at 110 rpm to facilitate biofilm formation [87]. Lids equipped with pegs were subjected to a sterile DBSP rinse (Gibco) for 5 min. Subsequently, the lid was placed onto a polypropylene 96-well plate (Corning 3879), which contained decreasing concentrations of peptide. This assembly was then incubated for an additional 24 h at 37 °C at 110 rpm. The staining of pegs using the crystal violet method followed previously reported procedures [45]. The pegs were washed thrice with cold tap water, heat-fixed at 70 °C for 1 h, and stained with a 0.1% crystal violet solution for 15 min. Excess stain was rinsed away with tap water and left to air-dry. The stained pegs were dissolved using 200 µL of 33% glacial acetic acid, and OD_590_ nm was measured to quantify the remaining biofilm. Pegs containing only bacteria were used as the growth control and represented 100% biofilm control. Controls for sterility included media alone and media supplemented with peptide. LL-37, IDR-1018, and polymyxin B were used as positive controls [40,41,42]. The experiment was performed twice. MBEC_50_ values were calculated by fitting the data from the MBEC assay (Figure S2) to a standard sigmoidal dose–response curve. For CFU log reduction determination, the manufacturer’s protocol was followed. The lid was rinsed once with sterile DPBS after incubation with the challenge. The lid was transferred into a new 96-well plate containing 200 µL of D/E neutralizing broth (BD 281910, Franklin Lakes, NJ, USA), and the plate was sonicated for 30 min to dislodge the biofilm. The plate with pegs containing biofilms was sonicated on high for 30 min (dry sonication) following Innovotech’s MBEC protocol [44,86]. The dry sonication was achieved by placing the MBEC plate in a stainless steel insert that sits on the water tray inside the 2.1 qt SP AmericanBrand Ultrasonic Sonic Cleaner/Bath Model C6450-46. After that, each well was serially diluted with DPBS and spotted on Tryptic Soy agar plates for colony counting.

### 4.8. Scanning Electron Microscopy of Biofilm

Visualization of bacteria was performed as previously described [88]. The biofilm was grown on pegs similar to the MBEC assay. Preformed biofilms were challenged with GATR-3. After incubation, the lid was transferred into a new 96-well plate containing 200 µL of Tryptic Soy broth (BD 211825), and the plate was sonicated for 30 min to dislodge the biofilm. The samples containing either bacteria alone or bacteria along with GATR3 were filttered using a 13 mm 0.2 µm polycarbonate filter (Whatman 10417001, Maidstone, UK) after the treatment of the membrane with 0.1% poly-L-lysine (Millipore Sigma P1274, St. Louis, MO, USA), to enhance bacterial adhesion [89]. Following this, the membranes housing the samples were fixed with 2.5% glutaraldehyde at 4 °C overnight. The samples were then dehydrated using a series of methanol concentrations (15%, 30%, 50%, 70%, 85%, and twice at 100%), with each concentration applied for 10 min per rinse. Subsequently, the samples were subjected to vacuum drying for 24 h. These membranes were affixed to specimen mounts using double carbon tape, sputter-coated, and observed utilizing a JSM-7200F field emission scanning electron microscope (JEOL USA, Inc., Peabody, MA, USA) at 15 kilovolts (kV).

### 4.9. Resistance Acquisition and Cross-Resistance

Sequential passaging of AB5075 with sub-MIC levels of GATR-3 and colistin (*n* = 3) in Difco MHB (BD 275730) was performed for 60 days to evaluate the development of resistance to GATR-3 and colistin following consistent exposure as previously reported [51]. Liquid cultures were initially challenged with subinhibitory peptide levels. Bacteria were grown in 1 mL of MHB for 24 h at 37 °C with shaking at 160 rpm. Daily, cultures that showed growth in the presence of challenge were diluted 1:100 in 1 mL fresh MHB with a determined challenge concentration for subsequent growth under the described conditions. Cultures were stored at −80 °C for MIC experiments by storing 1 mL at 0.5 McFarland in MHB with the addition of 40% glycerol (20% glycerol final concentration). The MIC was determined against GATR-3 and colistin every five passages, as described previously [31]. The concentration of added challenge was determined as resistance was observed by MIC. Colistin is reported to induce resistance within 5–7 days for *A. baumannii* [51]. Cross-resistance to GATR-3 was determined by determining the MIC of GATR-3 against colistin-exposed AB5075 (passage 60).

### 4.10. Membrane Disruption Assay

Membrane disruption evaluation was conducted by assessing ethidium bromide uptake after peptide exposure, following our previously established protocols [24,39,40,55,56,57,59,65,90]. AB5075 bacteria were cultured on Tryptic Soy agar for 18–24 h. Colonies displaying an opaque morphology were selected and suspended in DPBS (Gibco) to achieve an optical density (OD_600_) of 0.1. An aliquot of 180 µL of the bacterial suspension was combined to achieve a final peptide concentration of 50 µg/mL and a final ethidium bromide concentration of 10 µM within a black 96-well flat plate (Ultracruz Poly-propylene Microplate sc-204462, Santa Cruz, CA, USA). The plate was immediately transferred to a BioTek Cytation 5 instrument for fluorescence intensity measurement every 2 min for a duration of 20 min at 37 °C (with excitation at 535 nm and emission at 590 nm wavelengths). Relative fluorescence units (RFU) were calculated as follows: RFU of tested samples minus RFU of the no-peptide control containing ethidium bromide. LL-37 and polymyxin B were employed as controls in this experimental setup. The experiment was replicated three times and performed twice. Statistical significance was determined using Student’s *t*-test.

### 4.11. Membrane Depolarization Assay

The membrane depolarization assessment utilized the cationic dye, 3,3′-dipropylthiadicarbocyanine iodide (DiSC_3_(5)), following a modified version of our previously established method [24,55,56,65,91]. In brief, AB5075 bacterial colonies were cultured overnight on Tryptic Soy agar, and opaque colonies were dispersed in Dulbecco’s Phosphate-Buffered Saline (DPBS, Gibco) until reaching an optical density equivalent to 0.5 on the McFarland standard (~1 × 10^8^ CFU/mL). A bacterial suspension of 4 × 10^7^ CFU/mL was prepared, washed twice with DPBS, and subsequently resuspended in DPBS containing 10 µg/mL DiSC_3_(5). A 100 µL aliquot of the bacteria-DiSC_3_(5) suspension (*n* = 3) was dispensed into each well of a black 96-well flat plate (Ultracruz Poly-propylene Microplate sc-204462, Santa Cruz, CA, USA). The plate was incubated at room temperature within a BioTek Cytation 5 instrument and monitored until the fluorescence intensity stopped decreasing. Following this, a 100 µL aliquot of either peptide (final concentration of 50 µg/mL) or DPBS (untreated control) was added into each well, and the plate was promptly returned to the plate reader. Fluorescence readings were captured at 15-s intervals for a duration of 20 min (with excitation at 622 nm and emission at 670 nm). The experiment was conducted twice to ensure reproducibility of results.

### 4.12. Hemolysis Assay

The hemolytic activity of peptides was assessed by using a standardized protocol [23,24] adapted from [92]. Human red blood cells (RBCs) at a concentration of 2% were added to varying dilutions of peptides reconstituted in PBS within a sterile U-bottom polypropylene 96-well plate. Defibrinated human blood sourced from deidentified healthy donors from BioIVT (Westbury, NY, USA) was utilized. A volume of 1 mL of blood was centrifuged at 1600 g for 10 min to discard the plasma, followed by washing the RBCs at least thrice with sterile 1× PBS (HyClone, Marlborough, MA, USA). In the final wash step, the supernatant was removed, and the RBC pellet was resuspended in 750 µL of sterile PBS. A 2% RBC suspension was prepared by combining 200 µL of washed RBCs with 9.8 mL of sterile 1× PBS. After that, 50 µL of the 2% RBC suspension was added to each well (*n* = 3) containing diluted peptides, resulting in final peptide concentrations of 1, 10, 50, 100, 500, 1000, 2500, and 5000 µg/mL. Negative controls comprised 2% RBCs with 1× PBS alone (no peptide), while positive controls involved 2% RBCs in Triton X-100. The plate was incubated at 37 °C for 1 h, followed by centrifugation at 1000 rpm for 2 min. The supernatant was then transferred to a fresh 96-well plate (tissue culture-treated Falcon 353072) and assessed at OD540. The hemolysis percentage was determined relative to a 100% hemolysis control (Triton X-100). The experimental procedure was replicated twice for validation purposes.

### 4.13. Cell Cytotoxicity (MTT) Assay

Human HepG2 liver epithelial (HB-8065) cells were obtained from American Type Culture Collection, Ltd. (Manassas, VA, USA) and cultured in DMEM and Dulbecco’s modified Eagle’s medium (ATCC 30-2002) supplemented with FBS and fetal bovine serum (10% *v*/*v*) per manufacturer’s instructions. The cells were seeded in triplicate in 96-well plates and incubated at 37 °C in a 5% CO_2_ atmosphere. The Invitrogen CyQUANT MTT Cell Proliferation Assay Kit was used for the MTT assay. Cells were seeded at a cell density of 10,000 cells per well in a 96-well plate. The cells were incubated for 24 h for adherence. After 24 h, the reconstituted peptides at concentrations of 1 mg/mL, 10 mg/mL, and 100 mg/mL were added to each experimental well of the appropriate peptide in triplicate. The cells with peptide exposure were incubated for 24 h in CO_2_ at 37 °C. The controls included were the LL-37 peptide, IDR-1018 peptide, and “no treatment” samples. A 12 mM stock solution of MTT was made by adding 1 mL of sterile cell culture DPBS to 5 mg of MTT. The solution was vortexed until dissolved. After 24 h, the medium was removed from the adherent cells and replaced with 100 mL of Dulbecco’s modified Eagle’s medium (DMEM) with no phenol red. Ten milliliters of 12 mM MTT stock solution was added to each well and placed in an incubator protected from light for 4 h. After incubation, all but 25 mL of medium was removed from the wells. Next, 50 mL of 0.1 mM DMSO was added to each well and mixed thoroughly with a pipette. The plate was incubated for 10 min in the dark. Each sample was mixed thoroughly, and the samples were read at an absorbance of 540 nm. Cytotoxicity was obtained based on the values of the untreated controls.

### 4.14. Waxworm Toxicity Assessment

*Galleria mellonella* larvae were obtained from Vanderhorst Wholesale (Saint Mary’s, OH, USA). Ten larvae, each within the weight range of 200 to 300 mg, were assigned to individual experimental groups. To assess toxicity, an amount of 10 µg of GATR-3 dissolved in 10 µL of PBS was injected into the rear right proleg of each larva using a 250 µL Hamilton syringe equipped with a 30G needle (Hamilton, Reno, NV, USA). Subsequently, the survival of the larvae was monitored and recorded over a period of 72 h, following a previously established protocol [40,61]. The experiment was replicated twice, and the obtained data were analyzed using the Mantel–Cox test.

## Figures and Tables

**Table 1:** Apo6 and GATR-3 peptides properties. Apo6 and GATR-3 properties were calculated using the Antimicrobial Peptide Database (APD3) [27]. Amino acid substitutions are in bold. For I-Tasser structural prediction, C = coil, H = helix [26].

Property	Apo6	GATR-3
Peptide Sequence	KTRNWFSEHFKKVKEKLKDTFA	K**F**RNWFS**Q**HFKK**F**K**Q**KLK**N**TFA
I-Tasser Prediction	CCCHHHHHHHHHHHHHHHHCCC	CHHHHHHHHHHHHHHHHHHCCC
Peptide length	22	22
Net charge	+4	+7
Molecular Weight	2768.193 Da	2859.354 Da

**Table 2:** Minimum inhibitory concentration of GATR-3 peptide against a collection of MDR *A. baumannii* strains and other ESKAPE pathogens. The MIC is indicated in μg/mL. We followed the protocol for CLSI testing with the adaptation for peptides [28].

Peptide/Antibiotic	Organism	MIC (μg/mL)
GATR-3	*A. baumannii* AB5075 (MRSN 959)	4
	*A. baumannii* BAA-1710	
	*A. baumannii* BAA-1794	
	*A. baumannii* BAA-1795	
	*A. baumannii* BAA-1797	
	*A. baumannii* BAA-1799	
	*A. baumannii* BAA-1800	
	*A. baumannii* BAA-1605	
	*P. aeruginosa* BAA-2110	8
	*K. pneumoniae* BAA-1705	8
	*E. asburiae* BAA-3043	32
	*E. faecalis* 51299	64
	*MRSA* (*S. aureus* 33592)	64
Polymyxin B	*A. baumannii* AB5075	0.25–0.5
	*A. baumannii* BAA-1710	0.5
	*A. baumannii* BAA-1794	
	*A. baumannii* BAA-1795	
	*A. baumannii* BAA-1797	
	*A. baumannii* BAA-1799	
	*A. baumannii* BAA-1605	
	*A. baumannii* BAA-1800	0.5–1
Levofloxacin	*E. faecalis* 51299	0.5
Colistin	*K. pneumoniae* BAA-1705	0.5
Vancomycin	*S. aureus* 33592	2
Amikacin	*P. aeruginosa* BAA-2110	16–32
	*E. asburiae* BAA-3043	32

**Table 3:** Estimated MBIC_50_ of GATR-3, LL-37, IDR-1018 and polymyxin B against MDR *A. baumannii* strains.

Peptide/ Antibiotic	Organism	Approximate MBIC_50_ (µg/mL)	95% CI (µg/mL)
**GATR-3**	*A. baumannii* BAA-1794	0.62	0.3 to 1.2
	*A. baumannii* AB5075 (MRSN 959)	9.3	0.5 to 179.4
	*A. baumannii* BAA-1710	7.4	3 to 18.3
	*A. baumannii* BAA-1795	0.91	0.6 to 1.38
	*A. baumannii* BAA-1797	0.18	0.11 to 0.28
	*A. baumannii* BAA-1605	21.7	4.25 to 110.5
	*A. baumannii* BAA-1800	8.8	3.46 to 22.15
**LL-37**	*A. baumannii* BAA-1794	0.52	0.39 to 0.69
	*A. baumannii* AB5075 (MRSN 959)	>64	-
	*A. baumannii* BAA-1710	15.9	1.75 to 144.3
	*A. baumannii* BAA-1795	0.9	0.51 to 1.54
	*A. baumannii* BAA-1797	0.31	0.2 to 0.48
	*A. baumannii* BAA-1799	2	0.41 to 9.7
	*A. baumannii* BAA-1605	>64	-
	*A. baumannii* BAA-1800	>64	-
**IDR-1018**	*A. baumannii* BAA-1794	3.1	1.2 to 8.1
	*A. baumannii* AB5075 (MRSN 959)	>64	-
	*A. baumannii* BAA-1710	>64	-
	*A. baumannii* BAA-1795	11.2	4.8 to 26.00
	*A. baumannii* BAA-1797	0.68	0.32 to 1.4
	*A. baumannii* BAA-1799	>64	-
	*A. baumannii* BAA-1605	>64	-
	*A. baumannii* BAA-1800	48.7	17.8 to 133.3
**Polymyxin B**	*A. baumannii* BAA-1794	0.15	0.09 to 0.25
	*A. baumannii* AB5075 (MRSN 959)	0.27	0.09 to 0.82
	*A. baumannii* BAA-1710	1.21	0.21 to 6.8
	*A. baumannii* BAA-1795	0.46	0.21 to 1
	*A. baumannii* BAA-1797	0.08	0.056 to 0.12
	*A. baumannii* BAA-1799	0.96	0.2 to 4.5
	*A. baumannii* BAA-1605	1	0.63 to 1.6
	*A. baumannii* BAA-1800	0.9	0.34 to 2.37

**Table 4:** MBEC_50_ of peptides and antibiotics against tested bacteria.

Peptide/ Antibiotic	Organism	Estimated MBEC_50_ (µg/mL)	95% CI (µg/mL)
**GATR-3**	*A. baumannii* BAA-1794	9.6	4 to 23
	*A. baumannii* AB5075 (MRSN 959)	4.8	2.72 to 8.47
	*A. baumannii* BAA-1710	8	-
	*A. baumannii* BAA-1795	>64	-
	*A. baumannii* BAA-1797	6	2.85 to 12.8
	*A. baumannii* BAA-1605	4.4	2.42 to 8
	*A. baumannii* BAA-1800	4.1	2.72 to 6.2
**LL-37**	*A. baumannii* BAA-1794	2.97	1.2 to 7
	*A. baumannii* AB5075 (MRSN 959)	5.17	2.76 to 9.7
	*A. baumannii* BAA-1710	32–64	-
	*A. baumannii* BAA-1795	16–32	-
	*A. baumannii* BAA-1797	29.2	13.9 to 61
	*A. baumannii* BAA-1605	37.77	15.9 to 89.9
	*A. baumannii* BAA-1800	5.4	3.9 to 7.58
**IDR-1018**	*A. baumannii* BAA-1794	>64	-
	*A. baumannii* AB5075 (MRSN 959)	>64	-
	*A. baumannii* BAA-1710	>64	-
	*A. baumannii* BAA-1795	>64	-
	*A. baumannii* BAA-1797	>64	-
	*A. baumannii* BAA-1605	>64	-
	*A. baumannii* BAA-1800	>64	-
**Polymyxin B**	*A. baumannii* BAA-1794	0.18	0.1 to 0.32
	*A. baumannii* AB5075 (MRSN 959)	0.17	0.1 to 0.29
	*A. baumannii* BAA-1710	8	-
	*A. baumannii* BAA-1795	>32	-
	*A. baumannii* BAA-1797	0.29	0.13 to 0.7
	*A. baumannii* BAA-1605	0.53	0.32 to 0.89
	*A. baumannii* BAA-1800	0.29	0.19 to 0.42

**Table 5:** Calculation of the therapeutic index of GATR-3 against ESKAPE pathogens using MHC (95% CI) = 595 µg/mL (339–825) or estimated HC_50_ (µg/mL) = ~5 mg/mL.

Peptide	Organism	MIC (µg/mL)	MHC Therapeutic Index (TI)	HC_50_ Therapeutic Index (TI)
GATR-3	*A. baumannii*	4	149	1250
	*E. faecalis* 51299	64	9	78
	*S. aureus* 33592	64	9	78
	*K. pneumoniae* BAA-1705	8	74	625
	*P. aeruginosa* BAA-2110	8	74	625
	*E. asburiae* BAA-3043	32	19	156

## Data Availability

Data are contained within the article and Supplementary Materials.

## Supplementary Materials

The following supporting information can be downloaded at: https://www.mdpi.com/article/10.3390/antibiotics13010039/s1, Figure S1: Minimum inhibitory concentration (MIC) of GATR-3 against *A. baumannii*: AB5075, BAA-1710, BAA-1794, BAA-1795, BAA-1797, BAA-1799, BAA-1605 and BAA-1800; Figure S2: Minimum biofilm inhibitory concentration (MBIC) of GATR-3 against *A. baumannii*: AB5075, BAA-1710, BAA-1794, BAA-1795, BAA-1797, BAA-1605 and BAA-1800; Figure S3: Minimum biofilm inhibitory concentration (MBIC) of GATR-3 against *P. aeruginosa* BAA-2110, *K. pneumoniae*, *E. asburiae* BAA-3043, and *E. faecalis* 51299; Figure S4: Crystal violet staining of minimum biofilm eradication concentration (MBEC) assay. GATR-3 was challenged with *A. baumannii*: AB5075, BAA-1710, BAA-1794, BAA-1795, BAA-1797, BAA-1605 and BAA-1800; Figure S5: Sub-biofilm eradication concentration of GATR-3; Figure S6: Evaluation of cross-resistance development; Figure S7: Kinetics of membrane depolarization and disruption of GATR-3, LL-37 and polymyxin B (PMB) against AB5075 by the fluorescent dye DiSC_3_(5) and ethidium bromide; Figure S8: Hemolytic activity of GATR-3 and LL-37 against human red blood cells; Figure S9: GATR-3 peptide quality control information. Purity, liquid chromatography (HPLC), and mass-spectrometry (ESI-MS) analysis of GATR-3 peptides used in this study from the manufacturer; Figure S10: LL-37 peptide quality control information. Purity, liquid chromatography (HPLC), and mass-spectrometry (ESI-MS) analysis of LL-37 peptides used in this study from the manufacturers; Figure S11: IDR-1018 peptide quality control information. Purity, liquid chromatography (HPLC), and mass-spectrometry (ESI-MS) analysis of IDR-1018 peptides used in this study from the manufacturer. Table S1: List of MDR ESKAPE pathogens tested; Table S2: Sequences of peptides mentioned in this study; Table S3: Log of mean and standard deviation of MBEC assay.
