# Supplementary material for: GATR-3, a Peptide That Eradicates Preformed Biofilms of Multidrug-Resistant Acinetobacter baumannii
Source: Antibiotics (Basel). 2023 Dec 31;13(1):39. doi: 10.3390/antibiotics13010039 (PMC10812447; doi:10.3390/antibiotics13010039)

Supplementary Materials:

Table S1. List of MDR ESKAPE pathogens tested.

Strain	Source	Source information	MDR
Enterococcus (E.) faecalis 51299	ATCC	Human peritoneal fluid	VRE
S. aureus 33592	ATCC	Human blood	MRSA
K. pneumoniae BAA-1705	ATCC	Human urine	KPC
P. aeruginosa BAA-2110	ATCC	Human sputum	Yes
Enterobacter (E.) asburiae BAA-3043	ATCC	Human wound	Yes
A. baumannii AB5075 (MRSN 959)	BEI Resources	Human tibia/osteomyelitis	Yes
A. baumannii BAA-1710	ATCC	Human blood	Yes
A. baumannii BAA-1794	ATCC	Human sputum	Yes
A. baumannii BAA-1795	ATCC	Human nasotracheal aspirate	Yes
A. baumannii BAA-1797	ATCC	Human blood	Yes
A. baumannii BAA-1799	ATCC	Human blood	Yes
A. baumannii BAA-1605	ATCC	Human sputum	Yes
A. baumannii BAA-1800	ATCC	Human deep trachea	Yes

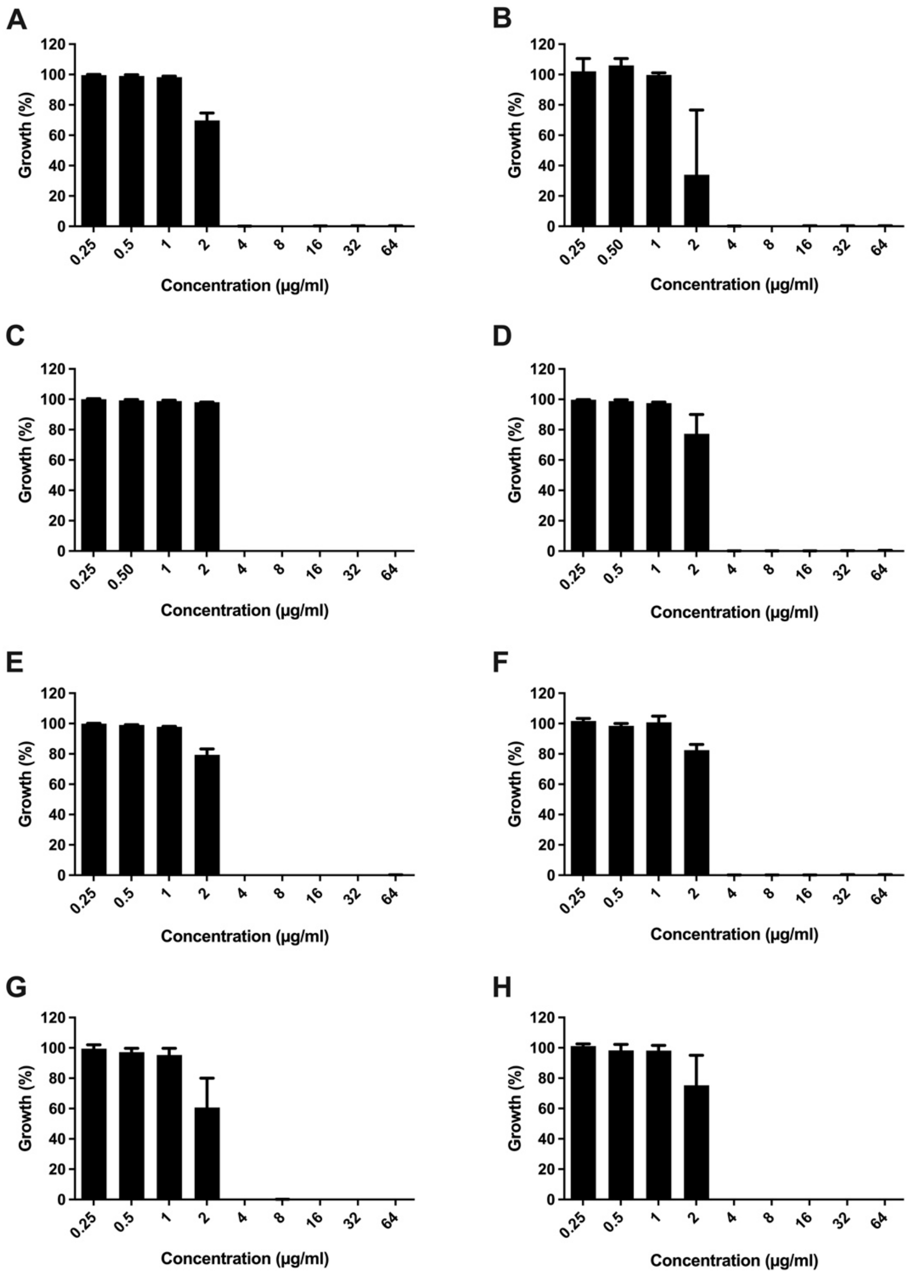

Figure S1. Minimum inhibitory concentration (MIC) of GATR-3 against *A. baumannii*: (**A**) AB5075, (**B**) BAA-1710, (**C**) BAA-1794, (**D**) BAA-1795, (**E**) BAA-1797, (**F**) BAA-1799, (**G**) BAA-1605 and (**H**) BAA-1800. Growth was measured spectrophotometrically (OD_{600}) after 20 – 24 h incubation. The percentage of growth was calculated relative to the untreated bacteria control (100%).

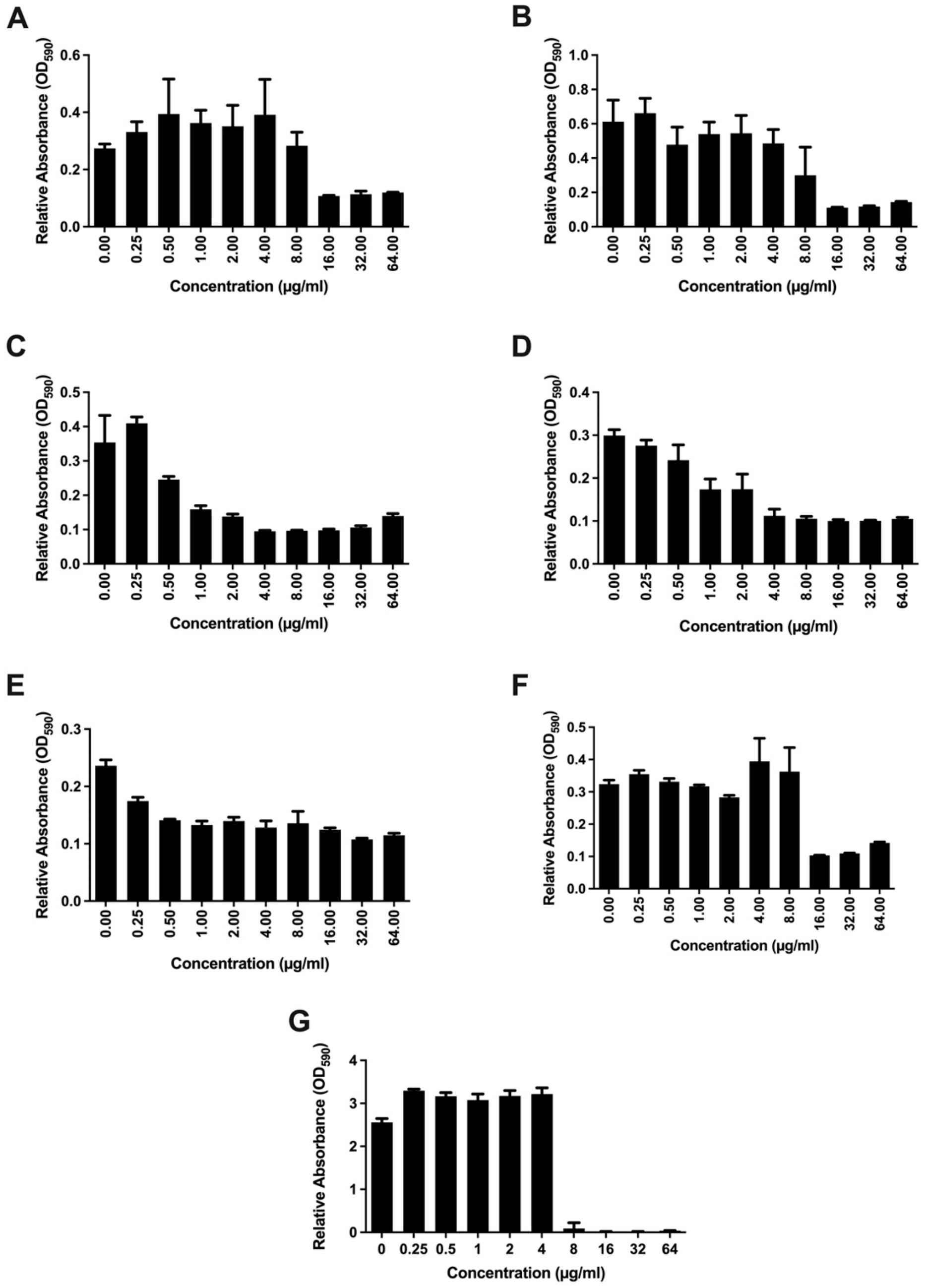

Figure S2. Minimum biofilm inhibitory concentration (MBIC) of GATR-3 against *A. baumannii*: (**A**) AB5075, (**B**) BAA-1710, (**C**) BAA-1794, (**D**) BAA-1795, (**E**) BAA-1797, (**F**) BAA-1605 and (**G**) BAA-1800. Crystal violet stain was read via OD_{590}.

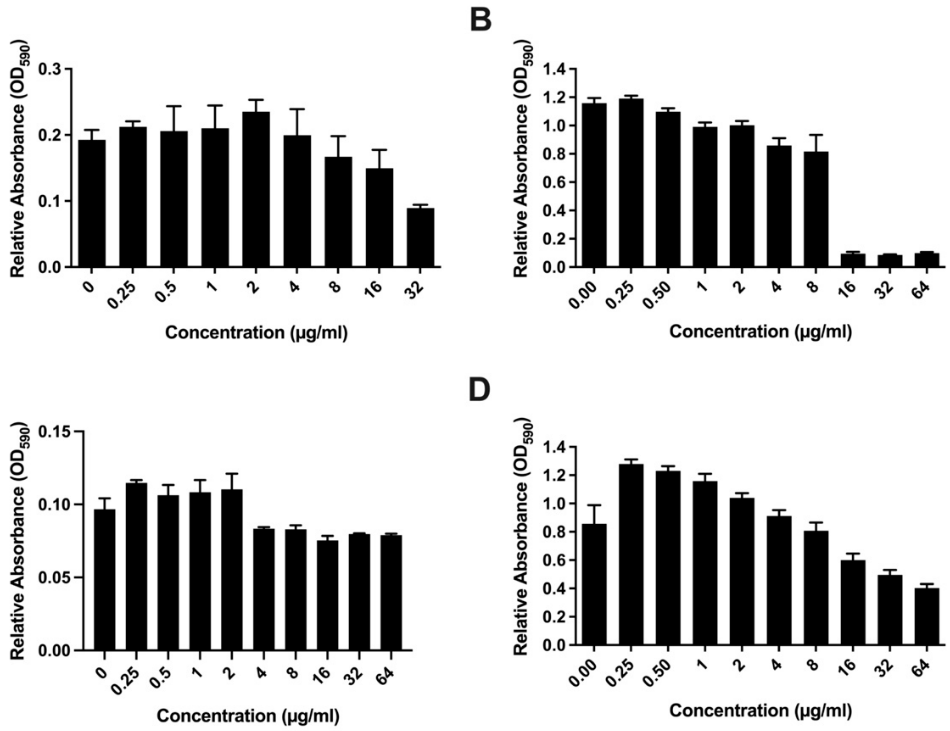

Figure S3. Minimum biofilm inhibitory concentration (MBIC) of GATR-3 against (**A**) *P. aeruginosa* BAA-2110, (**B**) *K. pneumoniae*, (**C**) *E. asburiae* BAA-3043, and (**D**) *E. faecalis* 51299. Crystal violet stain was read via OD_{590}.

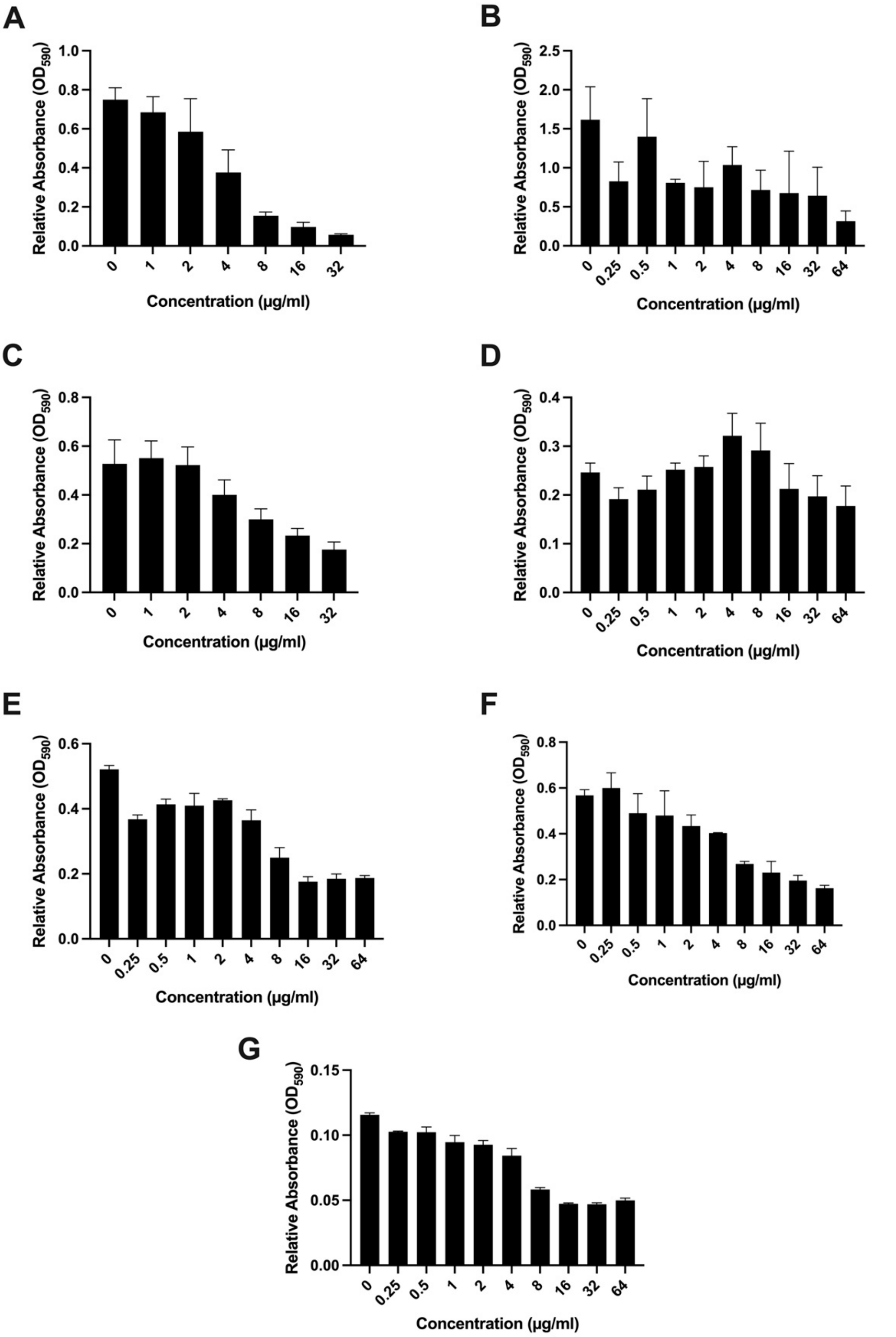

Figure S4. Crystal violet staining of minimum biofilm eradication concentration (MBEC) assay. GATR-3 was challenged with *A. baumannii*: (**A**) AB5075, (**B**) BAA-1710, (**C**) BAA-1794, (**D**) BAA-1795, (**E**) BAA-1797, (**F**) BAA-1605 and (**G**) BAA-1800. Crystal violet stain was read via OD_{590}.

Table S2. Sequences of peptides mentioned in this study.

Peptide	Sequence	Reference
HRZN-15	FLPWISKFLGKIL	[39]
SAAP-148	LKRVWKRVFKLLKRYWRQLKKPVR	[54]
LL-37	LLGDFFRKSKEKIGKEFKRIVQRIKDFLRNLVPRTES	[105]
Pexiganan	GIGKFLKKAKKFGKAFVKILKK	[106]

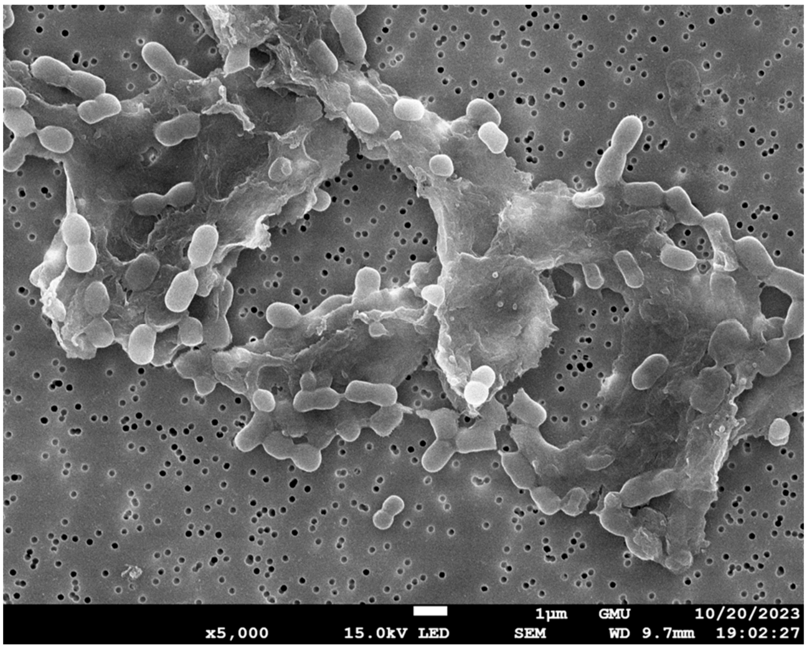

Figure S5. Sub-biofilm eradication concentration of GATR-3. Scanning electron micrograph of *A. baumannii* AB5075 treated with 16 µg/ml GATR-3 (in the treatment well). The image is taken at 5,000x with a working distance of 9.7 mm. The white bar represents 1 µm.

Table S3. Log of mean and standard deviation of MBEC assay.

**GATR-3**			**Polymyxin B**		
**Concentration µg/ml**	**Mean**	**SD**	**Concentration µg/ml**	**Mean**	**SD**
0	8.253	0.048	0	8.516	0.174
2	7.637	0.012	0.25	7.662	0.093
4	7.563	0.036	0.5	7.801	0.089
8	7.263	0.219	1	7.354	0.154
16	6.249	0.228	2	0	0
32	0	0	4	0	0
64	0	0	8	0	0
-	-	-	16	0	0

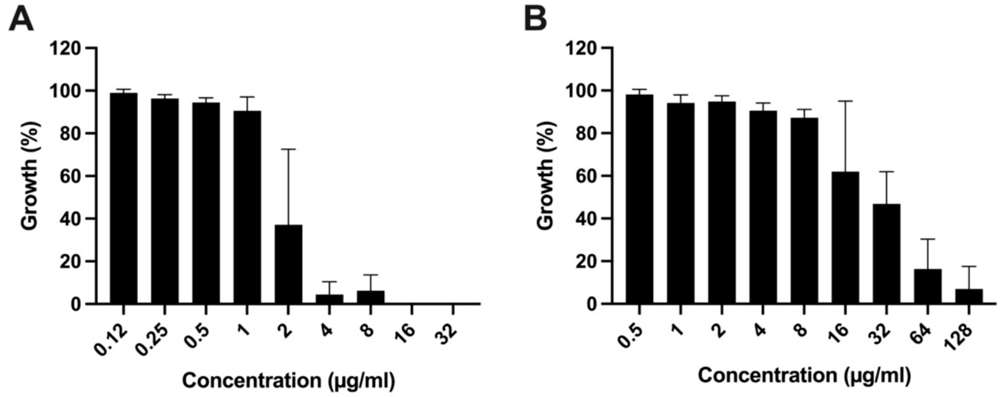

Figure S6. Evaluation of cross-resistance development of (**A**) GATR-3 against colistin-exposed *A. baumannii* from the 60th passage by minimum inhibitory concentration (MIC) assay. (**B**) Colistin was tested to show that the bacteria are resistant to colistin.

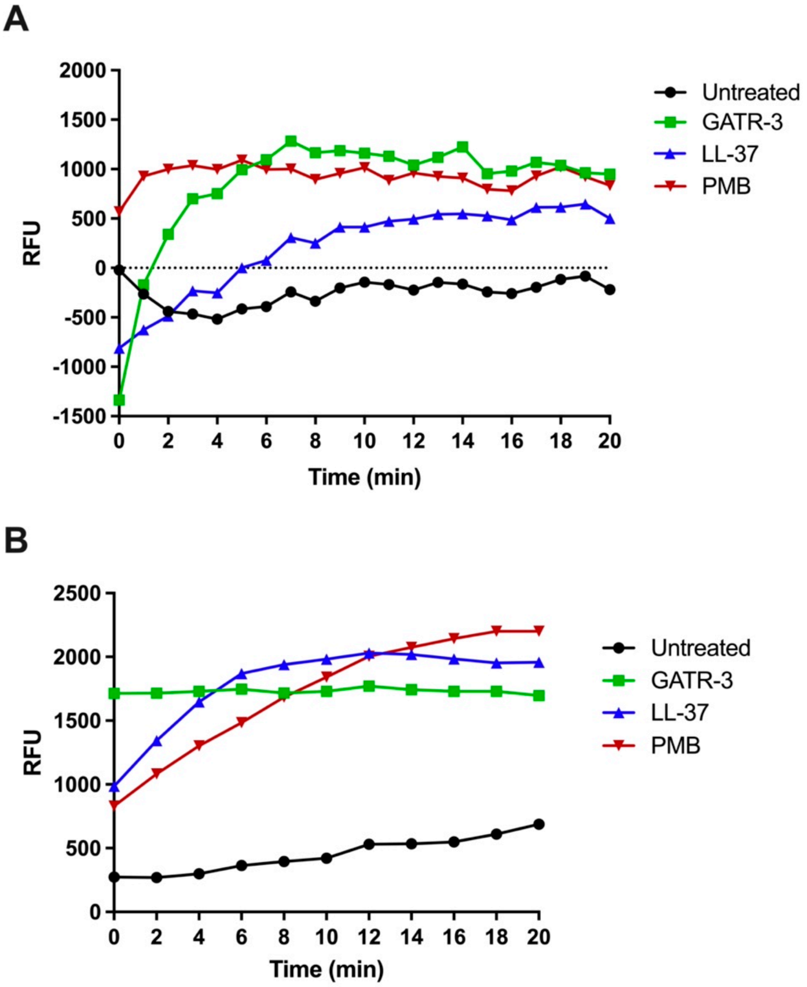

Figure S7. Kinetics of membrane depolarization (**A**) and disruption (**B**) of GATR-3, LL-37 and polymyxin B (PMB) against AB5075 by the fluorescent dye $DiSC_3(5)$ and ethidium bromide. Bacteria were incubated with 50 µg/ml peptide/drug, and the results were obtained after 20 minutes of challenge exposure.

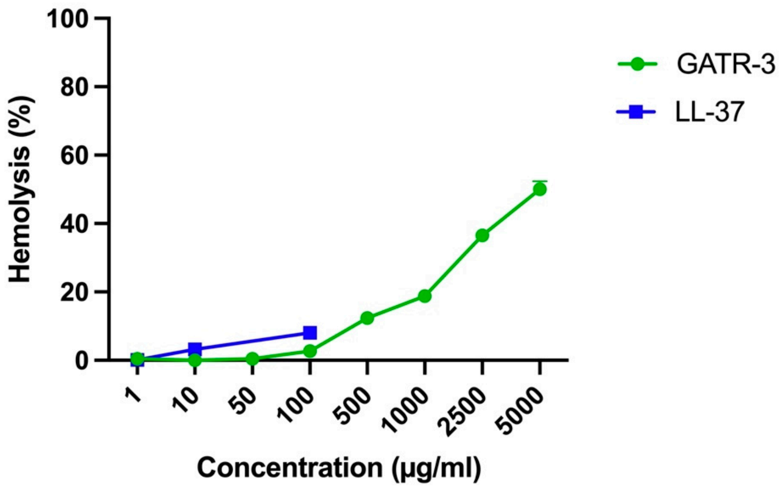

Figure S8: Hemolytic activity of GATR-3 and LL-37 against human red blood cells. A 2% RBC suspension was incubated with 5000, 2500, 1000, 500, 100, 50, 10, and 1 µg/ml GATR-3 and 100, 10, and 1 µg/ml LL-37. The release of hemoglobin (n = 3) was measured at OD_{540}, and the percentage of hemolysis was calculated relative to the Triton X-100-treated RBCs (100% hemolysis).

Figure S9: GATR-3 peptide quality control information. Purity, liquid chromatography (HPLC) and mass-spectrometry (ESI-MS) analysis of GATR-3 peptides used in this study from manufacturer.

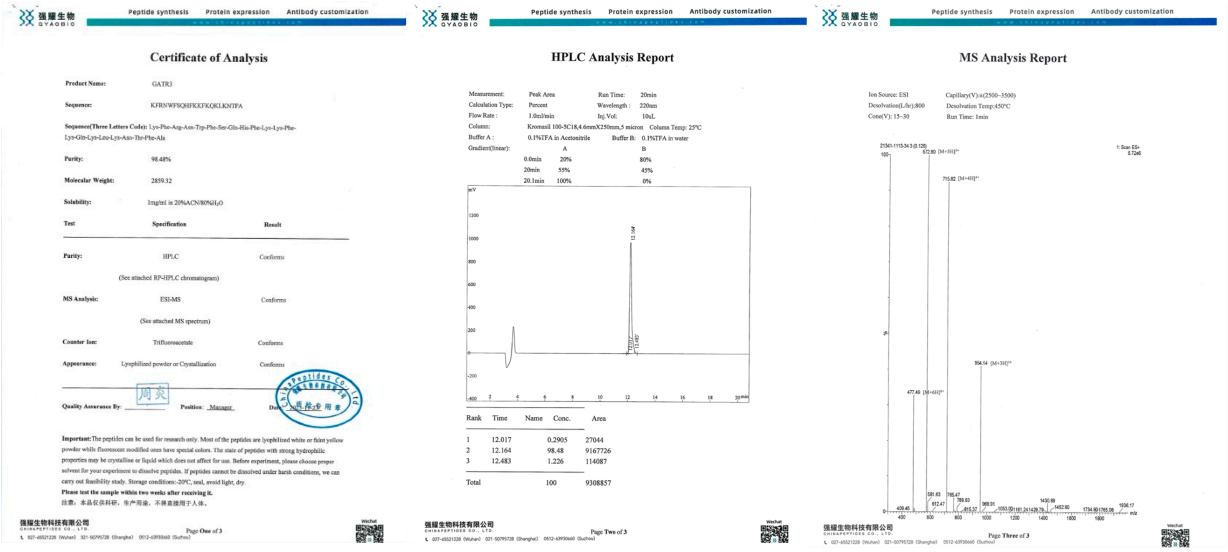

强耀生物 QYAOBIO — Peptide synthesis — Protein expression — Antibody customization — www.chinapeptides.com

Certificate of Analysis

Product Name:	GATR3	
Sequence:	KFRNWFSQHFKKFKQKLKNTFA	
Sequence(Three Letters Code):	Lys-Phe-Arg-Asn-Trp-Phe-Ser-Gln-His-Phe-Lys-Lys-Phe-Lys-Gln-Lys-Leu-Lys-Asn-Thr-Phe-Ala	
Purity:	98.48%	
Molecular Weight:	2859.32	
Solubility:	1mg/ml in 20%ACN/80%H_2O	

Test	Specification	Result
Purity:	HPLC (See attached RP-HPLC chromatogram)	Conforms
MS Analysis:	ESI-MS (See attached MS spectrum)	Conforms
Counter Ion:	Trifluoroacetate	Conforms
Appearance:	Lyophilized powder or Crystallization	Conforms

Quality Assurance By: 周炎 Position: Manager Date: 2023-[illegible]

Important: The peptides can be used for research only. Most of the peptides are lyophilized white or faint yellow powder while fluorescent modified ones have special colors. The state of peptides with strong hydrophilic properties may be crystalline or liquid which does not affect for use. Before experiment, please choose proper solvent for your experiment to dissolve peptides. If peptides cannot be dissolved under harsh conditions, we can carry out feasibility study. Storage conditions: -20°C, seal, avoid light, dry.

Please test the sample within two weeks after receiving it.

注意：本品仅供科研，生产用途，不得直接用于人体。

强耀生物科技有限公司 CHINAPEPTIDES CO., LTD. 027-65521228 (Wuhan) 021-50795728 (Shanghai) 0512-63930660 (Suzhou) Page One of 3 Wechat

HPLC Analysis Report

Measurement:	Peak Area	Run Time:	20min
Calculation Type:	Percent	Wavelength:	220nm
Flow Rate:	1.0ml/min	Inj.Vol:	10uL
Column:	Kromasil 100-5C18,4.6mmX250mm,5 micron	Column Temp:	25°C
Buffer A:	0.1%TFA in Acetonitrile	Buffer B:	0.1%TFA in water

Gradient(linear):	A	B
0.0min	20%	80%
20min	55%	45%
20.1min	100%	0%

Rank	Time	Name	Conc.	Area
1	12.017		0.2905	27044
2	12.164		98.48	9167726
3	12.483		1.226	114087
Total			100	9308857

强耀生物科技有限公司 CHINAPEPTIDES CO., LTD. 027-65521228 (Wuhan) 021-50795728 (Shanghai) 0512-63930660 (Suzhou) Page Two of 3 Wechat

MS Analysis Report

Ion Source: ESI	Capillary(V):±(2500~3500)
Desolvation(L/hr):800	Desolvation Temp:450°C
Cone(V): 15~30	Run Time: 1min

强耀生物科技有限公司 CHINAPEPTIDES CO., LTD. 027-65521228 (Wuhan) 021-50795728 (Shanghai) 0512-63930660 (Suzhou) Page Three of 3 Wechat

Figure S10: LL-37 peptide quality control information. Purity, liquid chromatography (HPLC) and mass-spectrometry (ESI-MS) analysis of LL-37 peptides used in this study from the manufacturers.

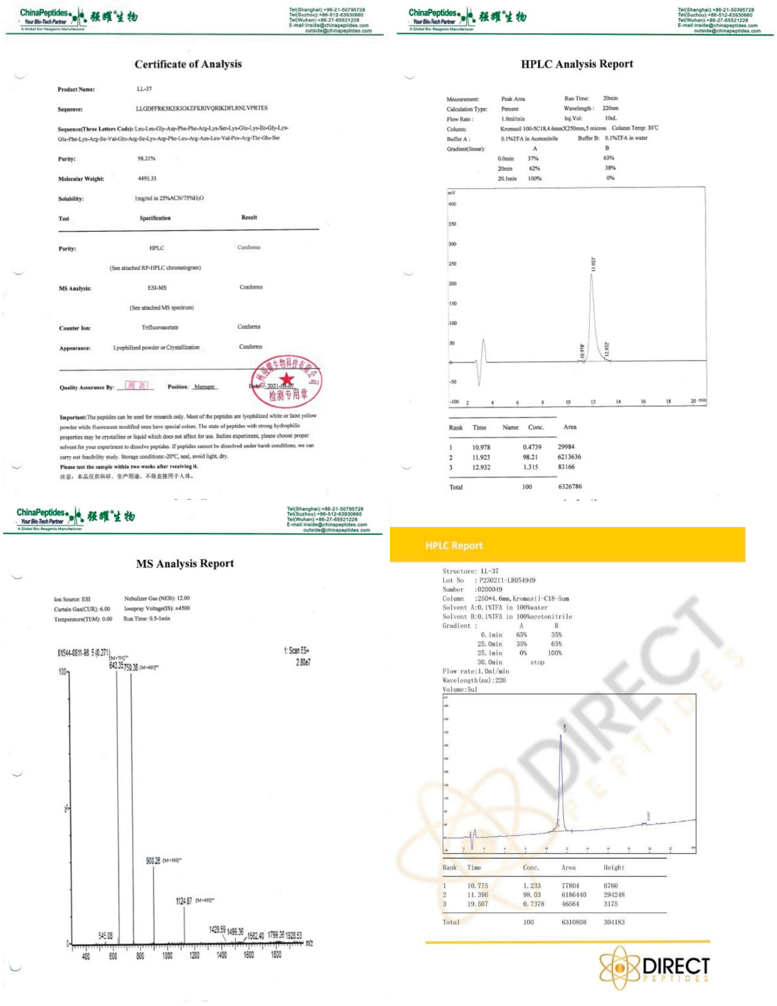

Figure S11: IDR-1018 peptide quality control information. Purity, liquid chromatography (HPLC) and mass-spectrometry (ESI-MS) analysis of IDR-1018 peptides used in this study from manufacturer.

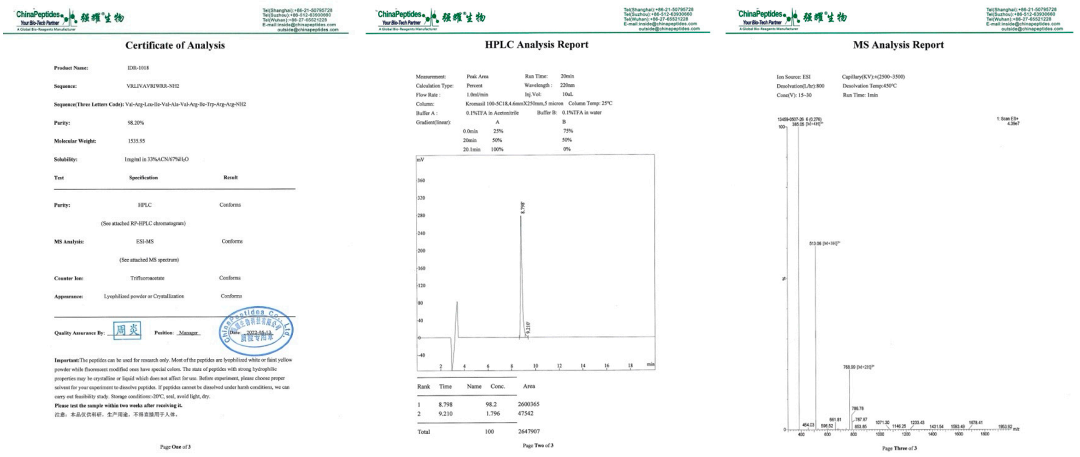

Supplement: Supplementary file 1 [file antibiotics-13-00039-s001.zip › antibiotics-2769535-supplementary.pdf]
